# A General Protein *O-*Glycosylation Gene Cluster Encodes the Species-Specific Glycan of the Oral Pathogen *Tannerella forsythia*: *O*-Glycan Biosynthesis and Immunological Implications

**DOI:** 10.3389/fmicb.2018.02008

**Published:** 2018-08-28

**Authors:** Markus B. Tomek, Daniel Maresch, Markus Windwarder, Valentin Friedrich, Bettina Janesch, Kristina Fuchs, Laura Neumann, Irene Nimeth, Nikolaus F. Zwickl, Juliane C. Dohm, Arun Everest-Dass, Daniel Kolarich, Heinz Himmelbauer, Friedrich Altmann, Christina Schäffer

**Affiliations:** ^1^NanoGlycobiology Unit, Department of NanoBiotechnology, Universität für Bodenkultur Wien, Vienna, Austria; ^2^Division of Biochemistry, Department of Chemistry, Universität für Bodenkultur Wien, Vienna, Austria; ^3^Bioinformatics Group, Department of Biotechnology, Universität für Bodenkultur Wien, Vienna, Austria; ^4^Institute for Glycomics, Griffith University, Brisbane, QLD, Australia

**Keywords:** carbohydrate-active enzymes, glycosyltransferase, immunogenicity, locus for glycosylation, methyltransferase, periodontitis, S-layer

## Abstract

The cell surface of the oral pathogen *Tannerella forsythia* is heavily glycosylated with a unique, complex decasaccharide that is *O*-glycosidically linked to the bacterium’s abundant surface (S-) layer, as well as other proteins. The S-layer glycoproteins are virulence factors of *T. forsythia* and there is evidence that protein *O-*glycosylation underpins the bacterium’s pathogenicity. To elucidate the protein *O*-glycosylation pathway, genes suspected of encoding pathway components were first identified in the genome sequence of the ATCC 43037 type strain, revealing a 27-kb gene cluster that was shown to be polycistronic. Using a gene deletion approach targeted at predicted glycosyltransferases (Gtfs) and methyltransferases encoded in this gene cluster, in combination with mass spectrometry of the protein-released *O-*glycans, we show that the gene cluster encodes the species-specific part of the *T. forsythia* ATCC 43037 decasaccharide and that this is assembled step-wise on a pentasaccharide core. The core was previously proposed to be conserved within the *Bacteroidetes* phylum, to which *T. forsythia* is affiliated, and its biosynthesis is encoded elsewhere on the bacterial genome. Next, to assess the prevalence of protein *O-*glycosylation among *Tannerella* sp., the publicly available genome sequences of six *T. forsythia* strains were compared, revealing gene clusters of similar size and organization as found in the ATCC 43037 type strain. The corresponding region in the genome of a periodontal health-associated *Tannerella* isolate showed a different gene composition lacking most of the genes commonly found in the pathogenic strains. Finally, we investigated whether differential cell surface glycosylation impacts *T. forsythia*’s overall immunogenicity. Release of proinflammatory cytokines by dendritic cells (DCs) upon stimulation with defined Gtf-deficient mutants of the type strain was measured and their T cell-priming potential post-stimulation was explored. This revealed that the *O-*glycan is pivotal to modulating DC effector functions, with the *T. forsythia*-specific glycan portion suppressing and the pentasaccharide core activating a Th17 response. We conclude that complex protein *O-*glycosylation is a hallmark of pathogenic *T. forsythia* strains and propose it as a valuable target for the design of novel antimicrobials against periodontitis.

## Introduction

Protein glycosylation in bacteria is a frequent modification of secreted and cell-surface proteins, such as flagella, pili, autotransporters, and surface (S-) layer proteins (Upreti et al., 2003; Schäffer and Messner, 2017). The biological roles of these glycoproteins strongly depend on the bacteria’s environmental context and cannot be predicted *a priori* (Varki et al., 2017). In several cases, general protein glycosylation systems are employed, yielding a suite of proteins with diverse locations and functionalities that carry one or more copies of an identical glycan (Schäffer and Messner, 2017). In bacterial genomes, the genetic information governing protein glycosylation is frequently organized in protein glycosylation gene clusters (Nothaft and Szymanski, 2010), which encode nucleotide sugar pathways genes, genes for Gtfs, glycan processing and modifying enzymes, ligases, and transporters. Based on our current knowledge, in bacteria, *O*-linked protein glycosylation (where the glycan is linked to Ser, Thr, or Tyr residues of the protein) seems to be more prevalent than *N*-linked protein glycosylation (where the glycan is bound to Asn) (Schäffer and Messner, 2017). Most protein *O-*glycosylation systems investigated so far secrete virulence factors or translocate glycoproteins to the bacterial cell surface, exemplified with *Campylobacter* spp. (Szymanski et al., 1999, 2003), *Neisseria* spp. (Ku et al., 2009; Vik et al., 2009; Hartley et al., 2011), *Bacteroides* spp. (Fletcher et al., 2009), *Actinomycetes* (Espitia et al., 2010), *Francisella tularensis* (Egge-Jacobsen et al., 2011), *Acinetobacter* spp. (Iwashkiw et al., 2012; Lees-Miller et al., 2013; Harding et al., 2015), *Burkholderia cepacia* (Lithgow et al., 2014), *Ralstonia solanacearum* (Elhenawy et al., 2016), and *Tannerella forsythia* (Posch et al., 2011).

**FIGURE 1:**
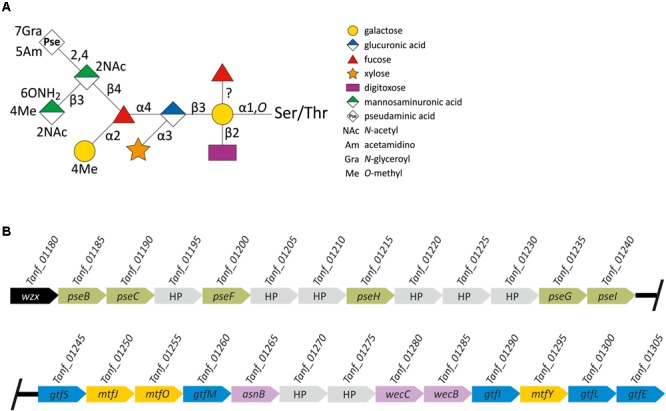
**(A)** Scheme of the *T. forsythia* ATCC 43037 S-layer *O*-glycan structure. Monosaccharide symbols are shown according to the Symbol Nomenclature for Glycans (SNFG) (Varki et al., 2015). Please note that the position of the branching Fuc remained unclear (Posch et al., 2011) until it was determined in the course of this study to be on the reducing-end Gal. **(B)** Scheme of the 27-kb protein *O*-glycosylation gene cluster of *T. forsythia* ATCC 43037. *Wzx* (black), flippase; *pseBCFHGI* (green), CMP-Pse biosynthesis genes; *gtfSMILE* (blue), Gtf genes; *mtfJOY* (yellow), Mtf genes; *asnB* (putative asparagine synthetase B), *wecC* (UDP-*N*-acetyl-D-mannosamine dehydrogenase) and *wecB* (UDP-*N-*acetylglucosamine 2-epimerase) (purple); hypothetical proteins, HP (gray). Genes are not drawn to scale.

*Tannerella forsythia* is a Gram-negative pathogen affiliated to the *Bacteroidetes* phylum of bacteria which utilizes a general protein *O*-glycosylation system to decorate several of its proteins with a so far unique, complex decasaccharide. The most abundant cellular proteins targeted by this system are the S-layer proteins TfsA and TfsB, which self-assemble on the bacterium’s cell surface into a two-dimensional crystalline monolayer (Sekot et al., 2012). The *T. forsythia O-*glycan (**Figure 1A**) is bound to distinct Ser and Thr residues within the three-amino acid motif D(S/T)(A/I/L/M/T/V) (Posch et al., 2011). The glycan is strain-specifically decorated with a modified terminal nonulosonic acid, which can be either a Pse as shown for the ATCC 43037 type strain, or a Leg exemplified by strain UB4 (Friedrich et al., 2017). Besides these sialic acid mimics, other unique S-layer glycan sugars present in the *T. forsythia O-*glycan are α-L-fucose (Fuc), Dig, Xyl, *N-*acetyl mannosaminuronic acid (ManNAcA), and *N*-acetyl mannosaminuronamide (ManNAcCONH_2_) (Posch et al., 2011).

To investigate the biosynthesis of the *T. forsythia O*-glycan, in a previous study, parts of an S-layer protein *O*-glycosylation gene locus were identified in the *T. forsythia* ATCC 43037 genome, and clustering of highly homologous genes was observed in different *Bacteroidetes* species (Posch et al., 2011). Based on that and a successful protein cross-glycosylation experiment between *T. forsythia* and *Bacteroides fragilis* (Posch et al., 2013), which both belong to the *Bacteroidetes* phylum, the presence of a phylum-wide protein *O-*glycosylation system was proposed (Coyne et al., 2013). An antiserum raised to a defined, truncated glycan of *B. fragilis* reacted with all *Bacteroidetes* species tested, including *T. forsythia*, but not with the full glycan, suggesting a discrimination between a core glycan and a species-specific glycan portion (Coyne et al., 2013).

In its native environment, *T. forsythia* thrives in a polymicrobial biofilm community that constitutes what is clinically described as oral plaque (Socransky et al., 1998; Holt and Ebersole, 2005). *Tannerella forsythia* is recognized as a key periodontal pathogen following the polymicrobial synergy and dysbiosis model of periodontal disease etiology (Hajishengallis and Lamont, 2012; Lamont and Hajishengallis, 2015). In this model, low abundance keystone pathogens are crucial as they initially subvert host immune responses, further leading to homeostasis breakdown in the oral cavity and destructive inflammation. Recent studies provided evidence that *T. forsythia* employs its unique cell surface to colonize its niche within the polymicrobial biofilm and to orchestrate the immune response of resident host tissue and the immune system (Bloch et al., 2017, 2018). In fact, the two S-layer glycoproteins (Sabet et al., 2003; Sharma, 2010; Sekot et al., 2011), as well as a leucine-rich repeat outer membrane glycoprotein BspA (Sharma et al., 2005), are among the bacterium’s virulence factors. The S-layer is strongly antigenic and mediates hemagglutination, as well as adherence to and invasion of human gingival epithelial cells (Sabet et al., 2003; Lee et al., 2006; Sakakibara et al., 2007). Studies with human macrophages and gingival fibroblasts demonstrated that the S-layer attenuates the host immune response by evading recognition by the innate immune system, at least at the early stage of infection (Sekot et al., 2011). There are indications that specifically the S-layer *O-*glycan is crucial for the modulation of host immunity through Th17 suppression (Settem et al., 2013). A very recent study suggests a role specifically of the modified Pse residue (Pse5Am7Gra) present at terminal position on the *T. forsythia* ATCC 43037 *O*-glycan in facilitating immune evasion by dampening the response of epithelial tissues to initial infection (Bloch et al., 2018). In addition to its immunological relevance, it was shown that the glycosylated *T. forsythia* S-layer plays a role in the bacterium’s biofilm life-style (Honma et al., 2007; Bloch et al., 2017; Friedrich et al., 2017).

These data indicate that the *T. forsythia O-*glycan and, hence, its biosynthesis pathway could be valuable targets in efforts to interfere with the establishment of periodontitis, which continues to be the most frequent inflammatory disease of bacterial origin world-wide (Hajishengallis, 2015). Thus, this present study was designed to obtain insight into the *T. forsythia O*-glycoprotein biosynthesis pathway and its involvement in underpinning the bacterium’s pathogenicity. Specifically, emphasis was focused on (i) sequence comparison of the general protein *O-*glycosylation gene cluster of the *T. forsythia* ATCC 43037 type strain with what is found in other *T. forsythia* strains for which genome sequences were available in public databases; (ii) transcriptional analysis of the protein *O-*glycosylation gene cluster of *T forsythia* ATCC 43037; (iii) construction of defined *T. forsythia* mutants deficient in predicted Gtfs and Mtfs encoded in the gene cluster and subsequent analysis of the *O-*glycans by MS to delineate the roles of the individual enzymes in glycan biosynthesis; and (iv) dissecting an *O-*glycan structure–function relationship in the immune response of DCs upon stimulation with the *T. forsythia* wild-type *versus* defined glycosylation-deficient mutants.

## Materials and Methods

### Bacterial Strains and Cultivation Conditions

*Tannerella forsythia* ATCC 43037 (American Type Culture Collection – ATCC, Manassas, VA, United States) (Tanner et al., 1986; Friedrich et al., 2015) and its mutants (**Table 1**) were grown anaerobically in brain heart infusion (BHI) media (Oxoid, Basingstoke, United Kingdom), supplemented with yeast extract (Sigma-Aldrich, Vienna, Austria), L-cysteine (Sigma-Aldrich), hemin (Sigma-Aldrich), menadione (Sigma-Aldrich), *N-*acetylmuramic acid (Carbosynth, Compton, United Kingdom), and horse serum (Thermo Fisher Scientific, Vienna, Austria) as described previously (Tomek et al., 2017). Media were supplemented with 50 μg/ml gentamicin, 5 μg/ml Erm or 10 μg/ml Cat, when appropriate.

**Table 1:** Bacterial strains and plasmids used in this study.

Strain or plasmid	Genotype and use or description	Source or reference
***Escherichia coli* strain**		
DH5α	F^-^ Φ80*lac*ZΔM15 Δ(*lac*ZYA-*arg*F) U169 *rec*A1 *end*A1 *hsd*R17 (rK–, mK+) *pho*A *sup*E44 λ- *thi*-1 *gyr*A96 *rel*A1; cloning strain	Invitrogen, Austria
***Tannerella forsythia* strains**		
ATCC 43037	Type strain, wild-type	ATCC; Friedrich et al., 2015
ATCC 43037 Δ*Tanf_01245*	Δ*Tanf_01245*::*ermF*; knock-out strain of *Tanf_01245*	Tomek et al., 2017
ATCC 43037 Δ*Tanf_01245^+^*	Δ*Tanf_01245*::*Tanf_01245 cat*; reconstituted knock-out strain	Tomek et al., 2017
ATCC 43037 Δ*Tanf_01250*	Δ*Tanf_01250*::*ermF*; knock-out strain of *Tanf_01250*	This work
ATCC 43037 Δ*Tanf_01255*	Δ*Tanf_01255*::*ermF*; knock-out strain of *Tanf_01255*	This work
ATCC 43037 Δ*Tanf_01260*	Δ*Tanf_01260*::*ermF*; knock-out strain of *Tanf_01260*	This work
ATCC 43037 Δ*Tanf_01260^+^*	Δ*Tanf_01260*::*Tanf_01260 cat*; reconstituted knock-out strain	This work
ATCC 43037 Δ*Tanf_01290*	Δ*Tanf_01290*::*ermF*; knock-out strain of *Tanf_01290*	This work
ATCC 43037 Δ*Tanf_01290^+^*	Δ*Tanf_01290*::*Tanf_01290 cat*; reconstituted knock-out strain	This work
ATCC 43037 Δ*Tanf_01295*	Δ*Tanf_01295*::*ermF*; knock-out strain of *Tanf_01295*	This work
ATCC 43037 Δ*Tanf_01300*	Δ*Tanf_01300*::*ermF*; knock-out strain of *Tanf_01300*	This work
ATCC 43037 Δ*Tanf_01300^+^*	Δ*Tanf_01300*::*Tanf_01300 cat*; reconstituted knock-out strain	This work
ATCC 43037 Δ*Tanf_01305*	Δ*Tanf_01305*::(P*erm*)-*ermF*; knock-out strain of *Tanf_01305*	This work
ATCC 43037 Δ*Tanf_01305^+^*	Δ*Tanf_01305*::*Tanf_01305 cat*; reconstituted knock-out strain	This work
**Plasmids**		
pJET1.2/blunt	Cloning vector; *amp^R^*	Thermo Fisher Scientific
pJET/ΔTF0955ko	Vector for amplification of the erythromycin resistance gene	Tomek et al., 2014
pEXALV	Vector for amplification of the chloramphenicol resistance gene	Zarschler et al., 2009
pJET1.2/Δ*Tanf_01250*	*Tanf_01250* knock-out cassette; *amp^R^ ermF^R^*	This work
pJET1.2/Δ*Tanf_01250^+^*	Cassette for reconstitution of Δ*Tanf_01250*; *amp^R^ cat^R^*	This work
pJET1.2/Δ*Tanf_01255*	*Tanf_01255* knock-out cassette; *amp^R^ ermF^R^*	This work
pJET1.2/Δ*Tanf_01260*	*Tanf_01260* knock-out cassette; *amp^R^ ermF^R^*	This work
pJET1.2/Δ*Tanf_01260^+^*	Cassette for reconstitution of Δ*Tanf_01260*; *amp^R^ cat^R^*	This work
pJET1.2/Δ*Tanf_01290*	*Tanf_01290* knock-out cassette; *amp^R^ ermF^R^*	This work
pJET1.2/Δ*Tanf_01290^+^*	Cassette for reconstitution of Δ*Tanf_01290*; *amp^R^ cat^R^*	This work
pJET1.2/Δ*Tanf_01295*	*Tanf_01295* knock-out cassette; *amp^R^ ermF^R^*	This work
pJET1.2/Δ*Tanf_01300*	*Tanf_01300* knock-out cassette; *amp^R^ ermF^R^*	This work
pJET1.2/Δ*Tanf_01300^+^*	Cassette for reconstitution of Δ*Tanf_01300*; *amp^R^ cat^R^*	This work
pJET1.2/Δ*Tanf_01305*	*Tanf_01305* knock-out cassette; *amp^R^ ermF^R^*	This work
pJET1.2/Δ*Tanf_01305^+^*	Cassette for reconstitution of Δ*Tanf_01305*; *amp^R^ cat^R^*	This work

*Escherichia coli* strains (**Table 1**) were grown under standard conditions in LB medium supplemented with 100 μg/ml ampicillin, when appropriate.

### Identification of a Protein *O-*Glycosylation Gene Cluster in *T. forsythia*

Up- and downstream regions of the previously identified protein *O-*glycosylation gene locus (Posch et al., 2011) were bioinformatically inspected using the genome sequence of the *T. forsythia* ATCC 43037 type strain as a basis (Friedrich et al., 2015). Information on putative gene functions were obtained through homology searches using the NCBI BLAST suite^1^ (Altschul et al., 1997) and the PFAM database^2^ (Finn et al., 2016).

An alignment of protein *O-*glycosylation gene clusters from different *T. forsythia* strains for which genome sequences were publically available was generated using the software MultiGeneBlast 1.1.13 (Camacho et al., 2009; Medema et al., 2013) in “homology search” mode. Genomes and annotations included in the analysis are listed in **Supplementary Table S1**. The genome assembly from *T. forsythia* FDC 92A2 was used as query, restricted to the genomic region from coordinates 1,135,922 to 1,166,078. The database was built from the assemblies of strains ATCC 43037, KS16, 3313, UB4, UB20, UB22 and *Tannerella* sp. HOT-286 (phylotype BU063). The assemblies were downloaded as GenBank flat files (gbff) from the NCBI ftp server^3^. RefSeq assemblies were downloaded for genomes FDC 92A2, ATCC 43037, KS16, 3313 and BU063. For strains UB4, UB20, and UB22, GenBank assemblies containing annotations generated in the context of the initial characterization of these genomes were used (Stafford et al., 2016). The RefSeq gbff files had to be adapted in order to provide unique protein identifiers in regions of interest as input for MultiGeneBlast. Minimal sequence identity (-minpercid) and minimal sequence coverage (-minseqcov) for protein alignments were both set to 50%, and default parameters -syntenyweight 0.5, -distancekb 20, -hitspergene 250 were used. For generating the BU063 track, an additional run with lower thresholds, i.e., 10% sequence identity and 10% sequence coverage, was performed. MultiGeneBlast runs were carried out under Linux Ubuntu 16.04; Linux shell commands were used for editing input files. The graphic files generated by MultiGeneBlast were combined and edited.

### RNA Purification and Reverse Transcription-PCR

Total RNA was extracted from *T. forsythia* ATCC 43037 using the PureLink RNA Mini Kit (Thermo Fisher Scientific) and subsequently treated with RNase-free DNase (PureLink DNase Set, Thermo Fisher Scientific) to remove DNA contamination. cDNA was generated using the MultiScribe Reverse Transcriptase from the High-Capacity cDNA Reverse Transcription Kit (Thermo Fisher Scientific) using 500 ng of total RNA. One tenth of each cDNA reaction mixture was used as template for PCR using Phusion High-Fidelity DNA polymerase (Thermo Fisher Scientific). The synthesized cDNA was amplified by PCR with primer pairs spanning stepwise neighboring genes, starting at the 5′ end of the gene cluster with *Tanf_01180*-*Tanf_01185* and ending with *Tanf_01300*-*Tanf_01305* (**Supplementary Figure S1**). Primer pairs and the size of the corresponding transcripts are indicated for each transcript in **Supplementary Figure S1** and primer sequences are listed in **Supplementary Table S2**. As a positive control, genomic DNA was used, and DNase treated RNA without the cDNA-generating step served as a control for contamination of total RNA with chromosomal DNA.

### Construction of Glycosyltransferase- and Methyltransferase-Deficient Mutants

Four putative Gtf genes (*Tanf_01260; Tanf_01290; Tanf_01300; Tanf_01305*) and three putative Mtf genes (*Tanf_01250; Tanf_01255; Tanf_01295*) encoded in the *T. forsythia* ATCC 43037 genome were individually knocked-out by homologous recombination, using a knock-out cassette deleting the selected gene. Clones viable on Erm-containing BHI-agar plates (0.8% w/v) were selected and further tested by PCR for correct integration of the knock-out cassette (**Supplementary Figures S2–S8**). A detailed description of the construction of the knock-out cassettes is given in the **Supplementary Material**. In short, approximately 1-kbp up- and downstream homology regions were joined to the Erm-resistance gene (P*ermF*, 1093-bp; *ermF*, 805-bp) by overlap-extension (OE)-PCR and subsequently blunt-end cloned into the plasmid pJET1.2 (Thermo Fisher Scientific). Knock-out cassettes were transformed into electrocompetent *T. forsythia* cells, which were regenerated overnight and plated on Erm-containing BHI selection plates. Single colonies were picked and used for inoculation of BHI medium. Once bacterial growth was visible, genomic DNA was isolated (Cheng and Jiang, 2006) and the loss of the selected gene (log) was tested by PCR (**Supplementary Figures S2–S8**). Phusion High-Fidelity DNA polymerase (Thermo Fisher Scientific) or Herculase II Fusion DNA Polymerase (Agilent Technologies, Vienna, Austria) were used for PCR amplification according to the manufacturer’s instructions. Oligonucleotides (Thermo Fisher Scientific) used are listed in **Supplementary Tables S3, S4**.

To exclude possible polar effects leading to an altered transcription of downstream genes, Gtf-deficient mutants were complemented with the native gene, in combination with a Cat resistance gene (chloramphenicol acetyl transferase, *cat;* 650 bp) for selection. For this purpose, the approximately 1-kbp homologous upstream region plus the associated native Gtf gene were joined to the *cat* resistance gene using OE-PCR and subsequently blunt-end cloned into the plasmid pJET1.2. Using the artificially introduced restriction sites SphI and KpnI, the approximately 1-kbp downstream homologous region was added, completing the reconstitution cassettes for *Tanf_01260* and *Tanf_01290* (**Supplementary Figures S2, S3**). The downstream region of the *Tanf_01300* reconstitution cassette was cloned via KpnI and NdeI restriction sites, while KpnI and BamHI were used for the *Tanf_01305* reconstitution cassette (**Supplementary Figures S4, S5**).

### SDS-PAGE and Western-Blotting

SDS-PAGE of crude cell extracts was carried out according to Laemmli (1970). 7.5% SDS-PA gels were prepared according to a standard protocol. Proteins were visualized with colloidal Coomassie Brilliant Blue (CBB) R-250 (Serva, Heidelberg, Germany) or transferred onto a polyvinylidene difluoride (PVDF) membrane (Bio-Rad, Vienna, Austria) for Western-blot analysis. Polyclonal rabbit antisera raised against the recombinant S-layer proteins TfsA (α-TfsA) and TfsB (α-TfsB) (Sekot et al., 2012) were used as primary antibodies in combination with a monoclonal goat α-rabbit secondary antibody labeled with IRDye 800CW (LI-COR Biosciences, Lincoln, NE, United States). S-Layer protein bands were visualized at 800 nm using an Odyssey Infrared Imaging System (LI-COR Biosciences).

### S-Layer *O*-Glycan Preparation and Liquid Chromatography ESI-MS

*O*-Glycans were released from the glycosylated S-layer proteins TfsA and TfsB by in-gel reductive β*-*elimination and purified by preparative PGC-HPLC as described previously (Posch et al., 2011; Tomek et al., 2014). The glycan mixture was analyzed using a Dionex Ultimate 3000 system directly linked to an ion trap instrument (amaZon speed ETD, Bruker, Germany) equipped with the standard ESI source in positive ion, Data Dependent Acquisition (DAA) mode (performing MS/MS on signals based on their intensity and LC elution). MS-scans were recorded over an *m*/*z* range 450–1650; ICC target was set to 100,000 and maximum accumulation time to 200 ms. The top 10 highest peaks were selected for fragmentation with an absolute intensity threshold above 50,000. Instrument calibration was performed using ESI Tuning Mix (Agilent Technologies) as by the manufacturer’s recommendations. LC separation of released *O-*glycans was performed on a Thermo Hypercarb separation column (5 μm particle size; 100 mm × 0.360 mm). A gradient from 1 to 21% solvent B in solvent A (solvent A, 65 mM ammonium formate buffer, pH 3.0; solvent B, 100% acetonitrile) over 20 min was applied, followed by a 10-min gradient from 21% B to 50% B, at a flow rate of 6 μl/min. Data were evaluated manually using the DataAnalysis 4.0 software (Bruker) and Glycoworkbench 2.1 build 146 (Ceroni et al., 2008).

### Generation of Human Monocyte- and Murine Bone Marrow-Derived Dendritic Cells

Human monocytes (E59; ∼40 × 10^6^ cells) from healthy volunteers (Ethical approval EK 1880/2012 in accordance with the Declaration of Helsinki, Medical University of Vienna, Vienna, Austria; written informed consent was obtained from all volunteers of this study) were thawed at 37°C and placed into AIM V medium (Gibco, Life Technologies, Paisley, United Kingdom) supplemented with 2% human plasma (Octaplas, OP; Octapharma, Zurich, Switzerland). Monocytes were counted and adjusted to a concentration of 0.5 × 10^6^ cells/ml. Dead cells were removed after incubation at 37°C for 1.5 h, while living cells were differentiated with 1000 U/ml GM-CSF and 400 U/ml IL-4. Medium including cytokines was replaced on day 3 and iDCs were harvested on day 5 (Lanzinger et al., 2012).

Murine BMDCs were isolated from C57BL/6 mice (Gallucci et al., 1999). Briefly, femurs and tibias were rinsed with DMEM, supplemented with 10% heat-inactivated fetal calf serum (FCS; PAA, Pasching Austria), 1% penicillin/streptomycin (Gibco), 1% non-essential amino acids (NEAA) (Gibco) and 0.0002% β-mercaptoethanol (Sigma-Aldrich). Erythrocytes were lysed in erolysis buffer (0.15 M NH_4_Cl, 10 mM KHCO_3_, 0.1 mM Na_2_EDTA; pH 7.2). The bone marrow was washed, and cells were re-suspended in supplemented DMEM. Cells were cultured in 24-well plates (Iwaki, Japan) at a concentration of 1.0 × 10^6^ cells per well in DMEM supplemented with 10% FCS and in the presence of 5 ng/ml recombinant murine IL-4 (eBioscience, San Diego, CA, United States) and 3 ng/ml recombinant murine GM-CSF (BD Pharmingen, San Diego, CA, United States). Half of the medium (including all supplements) was replaced every second day. Immature DCs (iDs) were harvested on day 6, counted and adjusted to a concentration of 1.0 × 10^6^ cells/ml. All mouse experiments were performed in accordance with the institutional guidelines and approved by the Animal Care and Use Committee of the Medical University of Vienna, Austria (GZ: 856861/2013/16).

### Stimulation of Dendritic Cells With Inactivated *T. forsythia* Cells

*Tannerella forsythia* wild-type and Gtf-deficient mutants were irradiated three times with UV light using maximum UV energy settings for 1 min, each, (Ultraviolet Crosslinker CL-1000; UVP, Upland, CA, United States) for inactivation. Cell integrity after treatment was verified by scanning electron microscopy using an Inspect S50 scanning electron microscope (FEI, Eindhoven, Netherlands) (Tomek et al., 2014).

Murine and human iDCs were matured in a 48-well plate with inactivated *T. forsythia* wild-type, Δ*Tanf_01245* (Δ*gtfS*), Δ*Tanf_01290* (Δ*gtfI*), or Δ*Tanf_01305* (Δ*gtfE*) cells, at concentrations of 10^6^, 10^7^ and 10^8^ cfu/ml. iDCs stimulated with 100 ng/ml LPS (*E. coli* strain O111:B4, Calbiochem, EMD Chemicals, San Diego, CA, United States) served as a positive control. Stimulated DCs were incubated at 37°C for 24 h. Supernatants were frozen for later use, DCs were harvested. DC maturation was monitored by visual and flow cytometric evaluation of typical DC morphology (LSR II flow cytometer, BD Biosciences), and expression of cell-surface markers (α-CD80, α-CD86, α-MHC-II; eBioscience) after 6 h and 24 h, respectively.

### Determination of Cytokine Levels by ProcartaPlex Multiplex Immunoassay and ELISA

Multiplex cytokine analysis of human monocyte-derived DC supernatants was performed using ProcartaPlex Multiplex immunoassay (eBioscience) for measuring cytokine secretion of IFN-γ, IL-1α, IL-1β, IL-4, IL-6, IL-10, IL-12p70, IL-23, and TNF-α.

The level of secreted cytokines in murine BMDC supernatants, TNF-α and IL-6, were analyzed using commercial ready-set-go ELISA kits (eBioscience) in 96-well microtiter plates according to the manufacturer’s protocol and measured at 450 nm by a micro plate reader (PerkinElmer, EnSpire Multimode Reader, Waltham, MA, United States).

### T Cell-Priming Upon Stimulation of Antigen-Presenting Cells With *T. forsythia*

Human PBMCs were separated from whole blood by density centrifugation (Lanzinger et al., 2012). PBMCs were cultured in RPMI-1640 medium supplemented with GlutaMAX–I (Gibco) and 2% human plasma (OP, Octapharma). PBMCs were counted with BD Trucount tubes (BD Biosciences), adjusted to a concentration of 1.0 × 10^7^ cells/ml and labeled with CFSE (Invitrogen, Austria). T cell priming upon antigen-presenting cell (APC) stimulation with *T. forsythia* wild-type and Gtf-deficient mutants was assessed in the mixed PBMC culture. Whole PBMCs stimulated with 50 ng/ml LPS (*E. coli* O111:B4 LPS, Merck, Darmstadt, Germany) and 10^3^ U/ml human recombinant IFN-γ (PeproTech, Rocky Hill, NJ, United States) served as positive controls. T cell proliferation was calculated as percentage CFSE negative cells of CD3^+^ cells after 8 days of co-culture (Lyons and Parish, 1994). T cell activation was measured via expression of CD25 (α-CD25; BD Pharmingen) by flow cytometry. CD4^+^ T cell differentiation (α-CD4; eBiosciences) was assessed by expression of signature transcription factors for Treg (FoxP3; BD Pharmingen) and Th17 (RORγT; eBiosciences) cells as measured by flow cytometry. Th1 differentiation was assessed by expression of the signature transcription factor T-bet (α-Tbet; eBiosciences) as measured by flow cytometry.

Data analysis was made using the Student’s *t*-test, as appropriate. A *p*-value below 0.05 was considered to indicate statistical significance.

## Results

### Genomic Organization of the General Protein *O*-Glycosylation Gene Cluster of the *T. forsythia* Type Strain

Previously, a partial protein *O-*glycosylation gene locus (Posch et al., 2011; Coyne et al., 2013) as well as genes for the biosynthesis of CMP-Pse, which is the activated form of this sugar acid required for its incorporation into the S-layer glycan, (Friedrich et al., 2017), were identified in the genome of *T. forsythia* ATCC 43037. That genomic region (Friedrich et al., 2015) was now investigated *in silico* for the presence of further genes with functional annotations as carbohydrate-active enzymes. This revealed a 27-kb protein *O-*glycosylation gene cluster (**Figure 1B**). From the 5′ end, the gene cluster starts with a *wzx* flippase-like gene (*Tanf_01180*) which is followed by the six genes required for the biosynthesis of CMP-Pse (Friedrich et al., 2017), present in the order *pseB* (*Tanf_01185*), *pseC* (*Tanf_01190*), *pseF* (*Tanf_01200*), *pseH* (*Tanf_01225*), *pseG* (*Tanf_01235*), *pseI* (*Tanf_01240*). ORFs encoding hypothetical proteins of unknown function (HP) intercept this region. Downstream, five genes encoding proteins with Gtf domains (*Tanf_01245, Tanf_01260, Tanf_01290, Tanf_01300, Tanf_01305*, named *gtfS, gtfM, gtfI, gtfL*, and *gtfE*) are present. The corresponding glycosyltransferases GtfS (445 amino acids), GtfM (382 amino acids), GtfI (402 amino acids; GT1 family of Gtfs) and GftL (420 amino acids; GT1 family of Gtfs) exhibit homology to the GT-B type superfamily of Gtfs, while GtfE (255 amino acids; GT2 family of glycosyltransferases) exhibits homology to the GT-A type superfamily.

Further downstream, WecC (Tanf_01280, UDP-*N*-acetylmannosaminuronic acid dehydrogenase) and WecB (Tanf_01285, UDP-*N-*acetylglucosamine 2-epimerase) are encoded, predicted to be involved in the biosynthesis of UDP-*N-*acetylmannosaminuronic acid. The conserved AnsB domain of Tanf_01265 (605 amino acids; predicted asparagine synthetase B) indicates amidotransferase activity of this ORF, putatively involved in the formation of the acetamidino (Am) modification on the C-5 at the Pse residue. A candidate gene for the glyceroyl (Gra) modification at the C-7 of Pse remains elusive. Additionally, within the protein *O-*glycosylation gene cluster, three genes were identified sharing similarities with SAM-dependent Mtfs; these are *Tanf_01250, Tanf_01255* and *Tanf_01295*, encoding proteins named MtfJ (90 amino acids), MtfO (167 amino acids), and MtfY (213 amino acids), respectively. SAM-dependent domains are present in all of these Mtfs; the typical glycine-rich sequence (motif I; E/DXGXGXG) (Martin and McMillan, 2002; Schubert et al., 2003) is only found within the amino acid sequence of MtfJ.

Inspection of eight further ORFs distributed over the entire protein *O-*glycosylation gene cluster, i.e., Tanf_01195, Tanf_01205, Tanf_01210, Tanf_01220, Tanf_01225, Tanf_01230, Tanf_01270, and Tanf_01275, did not provide evidence for functions related to carbohydrate metabolism. Thus, these genes were regarded as irrelevant for the present study.

### Transcription Analysis of the Protein *O*-Glycosylation Gene Cluster

To analyze whether the genes encoded by the protein *O-*glycosylation gene cluster are transcriptionally linked, total RNA from *T. forsythia* ATCC 43037 cells was extracted and co-transcription of the relevant genes was analyzed using RT-PCR as outlined in **Supplementary Figure S1**. PCR products of the expected sizes were obtained with primer pairs (**Supplementary Table S2**) designed to bridge the ends between the ORFs of adjacent genes, yielding amplification products only when co-transcription was occurring. The results revealed that the cluster is transcribed as a polycistronic unit spanning at least the *Tanf_01180* to *Tanf_01305* genes (**Supplementary Figure S1**). This implicates that a previously described three-gene “exopolysaccharide synthesis operon” spanning *Tanf_01280* to *Tanf_01290* (Honma et al., 2007) is part of the contiguous transcription unit of the *T. forsythia* ATCC 43037 protein *O-*glycosylation gene cluster.

### The General Protein *O-*Glycosylation Gene Cluster Is a Common Feature of Pathogenic *T. forsythia* Strains

The species-wide conservation of the general protein *O-*glycosylation gene cluster was assessed by sequence comparison of the ATCC 43037 type strain with six other publicly available *T. forsythia* genomes (*T. forsythia* UB20, FDC 92A2, UB4, KS16, UB22, and 3313; genome accessions are listed in **Supplementary Table S1**). Prior to that, it was confirmed by sequence homology searches, that all of these genomes possessed orthologs of the ATCC 43037 S-layer genes *tfsA* (*Tanf_03370*) and *tfsB* (*Tanf_03375*), which are the most abundant glycosylation targets in *T. forsythia* (**Supplementary Table S5**).

**FIGURE 2:**
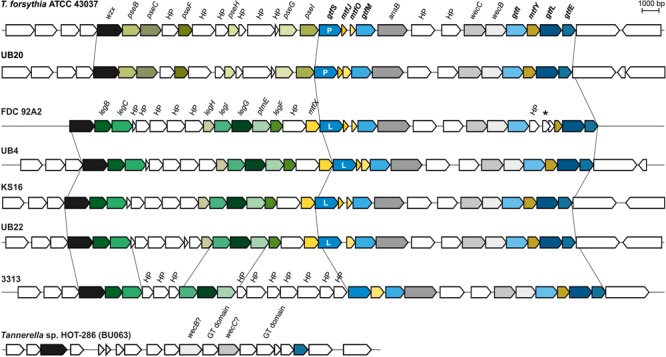
Alignment of protein *O-*glycosylation gene clusters from different *T. forsythia* strains showing comparable sizes and gene organizations (drawn to scale). Genes showing sequence identity > 50% and sequence coverage > 50% between strains appear in the same color. The major difference in the analyzed strains are for genes synthesizing either CMP-Pse (*pseBCFHGI*; light green colors; strains ATCC 43037 and UB20) or CMP-Leg (*legBCHIGF, ptmE*; dark green colors; strains FDC 92A2, UB4, KS16, UB22). Genes encoding Gtfs (*gtfSMILE*; blue color), Mtfs (*mtfJOY*; yellow color) and carbohydrate modifying enzymes (*asnB, wecC, wecB*; gray color) show high sequence homology between analyzed strains. Genomes of all strains synthesizing CMP-Leg encode an additional putative Mtf gene (*mtfX*), which does not share sequence homology to other Mtfs located within the cluster. In strain UB22, *mtfJ* is not predicted and for strain 3313 only five out of seven genes needed for the synthesis of CMP-Leg are predicted confidently. Due to low homology, isolate *Tannerella* sp. HOT-286 (phylotype BU063) could not be aligned with the other *T. forsythia* strains; for that isolate, the genomic area between a *wzx*-like gene and the *gtfE* gene is shown for comparison. P, Pse transferase; L, Leg transferase; HP, hypothetical protein; the star symbol (^∗^) indicates a transposable element; genes written in bold letters were investigated in detail in course of this study.

In all of the analyzed *T. forsythia* genomes, general protein *O*-glycosylation gene clusters of comparable size, content, and organization were identified (**Figure 2**). The major difference found between the analyzed gene clusters was in the alternate presence of six genes encoding the biosynthesis pathway for either CMP-Pse (*pseBCHGIF* in strains ATCC 43037 and UB20) or CMP-Leg (*legBCHGIF* in strains FDC 92A2, UB4, KS16, and UB22), respectively. In all cases, the corresponding genes are located immediately downstream of a conserved *wzx*-flippase like gene (*Tanf_01180* in strain ATCC 43037). Strain 3313 presents a unique situation, since most components of the CMP-Leg pathway were found, however, *legF* could not be identified and for *legH* only a match at 29% sequence identity (covering 95% of the sequence) could be detected. These data leave a prediction of the type of nonulosonic acid in this strain open, if present at all. Furthermore, 10 genes of unknown function that do not have counterparts in the other *T. forsythia* protein *O-*glycosylation gene clusters are present in that region of the 3313 genome. Apart from this difference, the region further downstream shares a high degree of similarity with the other protein *O-*glycosylation gene clusters. This region encodes five predicted glycosyltransferases (GtfSMILE), two UDP-*N*-acetylmannosaminuronic acid biosynthesis enzymes (WecB, WecC), and the putative carbohydrate modifying enzyme AsnB (**Figure 2**), as described above for *T. forsythia* ATCC 43037. With regard to the three Mtfs (MtfJOY) predicted for the type strain, no *mtfJ* gene was annotated in strain UB22. In strain 3313, a single gene was annotated at the location of *mtfJ* and *mtfO*, showing sequence homology to both *mtfJ* (93% of its length) and *mtfO* (entire length), suggesting either misannotation or a fused *mtfJ–mtfO* gene. All CMP-Leg synthesizing strains contained an additional predicted Mtf gene (named *mtfX*) which does not share sequence homology to the Mtfs MtfJOY. In strain FDC 92A2, there is a transposable element present between the *gtfI* and *mtfY* genes.

In contrast, the genome sequence of *Tannerella* sp. HOT-286 (phylotype BU063; genome accession NZ_CP017038.1), which is described as a periodontal health-associated isolate (Beall et al., 2014), showed sequence homology to only the *wzx* flippase-like gene (46% sequence identity) and the Gtf gene *gtfE* (69% sequence identity). Apart from weak matches to the *wecB* and *wecC* genes, there was no further homology to genes found in the protein *O-*glycosylation gene cluster of pathogenic *T. forsythia* strains. Instead, two genes with predicted Gtf domains (locus tags: BCB71_RS10435 and BCB71_RS10420), which are different from the *gtfSMILE* genes mentioned before, were found in that region of the HOT-286 (phylotype BU063) genome (**Figure 2**). Further research on this difficult-to-grow *Tannerella* sp. (Vartoukian, 2016) is needed to assess its glycosylation potential and potential role in periodontal health and disease.

### Glycosyltransferases of the Protein *O*-Glycosylation Gene Cluster Assemble the Species-Specific Glycan

**FIGURE 3:**
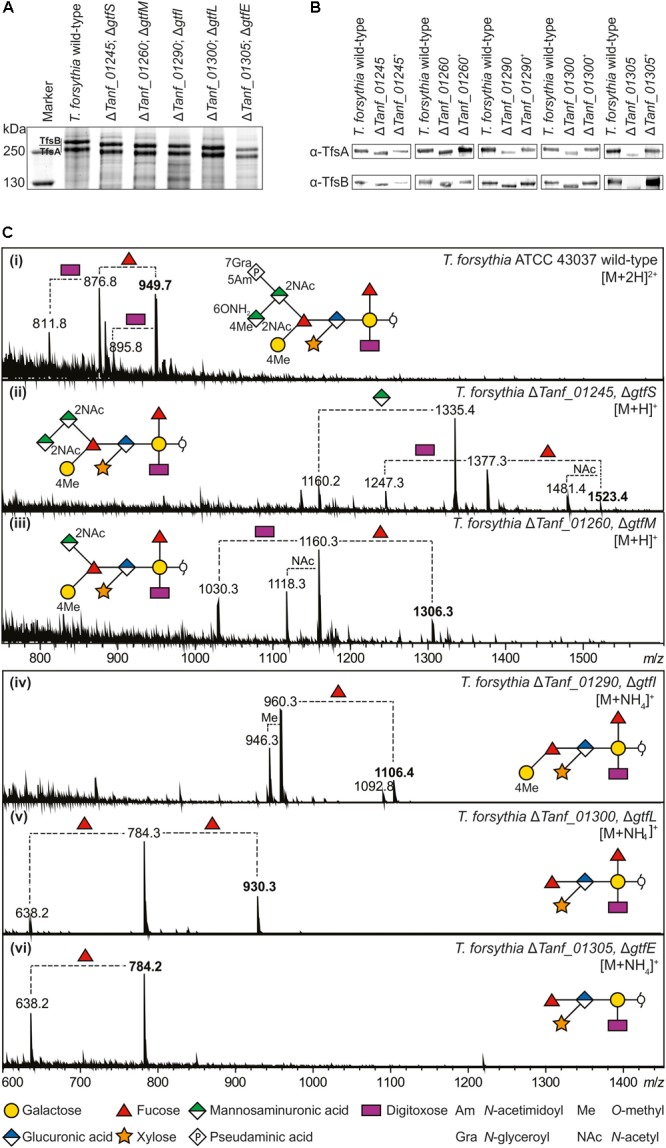
**(A)** Coomassie Brilliant Blue staining of crude cell extracts from *T. forsythia* ATCC 43037 wild-type and glycosyltransferase-deficient mutants after separation on a 7.5% SDS-PA gel. The S-layer glycoproteins (labeled TfsA and TfsB) are indicated and the downshifts resulting from glycan truncation can be seen in the mutants. S-layer glycoprotein bands were further processed for MS analyses. PageRuler Plus Prestained Protein Ladder (Thermo Fisher Scientific) was used as a protein molecular weight marker. **(B)** Western-blots probed with α-TfsA and α-TfsB antiserum for confirmation of the identity of S-layer glycoproteins. Glycoproteins from all glycosyltransferase-deficient mutants (Δ*gtfSMILE*) experienced a downshift resulting from glycan truncation, whereas the reconstituted strains (denoted with +) regained wild-type migration, indicating the presence of the complete mature glycan, proving successful recombination. **(C, i–vi)** ESI-MS sum spectra of β-eliminated TfsB *O*-glycans from *T. forsythia* wild-type and mutants. The glycan structures of the signals corresponding to the largest mass (bold *m*/*z* values) are shown in SNFG representations (Varki et al., 2015). *O*-Glycan signals detected for the respective mutants were assigned based on the *m*/*z* mass differences corresponding to the loss of individual sugar units and/or modifications.

To investigate the principal involvement of the predicted Gtfs GtfSMILE in the biosynthesis of the *T. forsythia* ATCC 43037 S-layer *O-*glycan, single gene knock-out mutants of the corresponding genes, i.e., *Tanf_01260* (*gtfM*), *Tanf_01290* (*gtfI*), *Tanf_01300* (*gtfL*) and *Tanf_01305* (*gtfE*), were created (**Supplementary Figures S2–S5**) - Δ*Tanf_01245* (Δ*gtfS*) was available from a previous study (Tomek et al., 2017) - and analyzed by SDS-PAGE and Western-blotting. In comparison to the *T. forsythia* ATCC 43037 wild-type, in which the glycosylated S-layer proteins TfsA and TfsB migrate on the SDS-PAGE gel at ∼230 kDa (calculated MW of the protein, 135 kDa) and ∼270 kDa (calculated MW of the protein, 152 kDa), respectively, each Gtf-deficient mutant experienced a downshift of these prominent *T. forsythia* glycoproteins (**Figure 3A**). Downshifted S-layer glycoproteins suggest a stepwise truncation of the *O-*glycan from wild-type via Δ*gtfS*, Δ*gtfM*, Δ*gtfI*, Δ*gtfL* to Δ*gtfE*. Western-blots probed with α-TfsA and α-TfsB antiserum confirmed the identity of the S-layer glycoproteins in *T. forsythia* wild-type and mutant strains, with the S-layer glycoproteins of the reconstituted mutants regaining their native SDS-PAGE migration profile (**Figure 3B**). MS analysis of β-eliminated TfsB *O-*glycans from the Gtf mutants upon complementation with the native gene (*T. forsythia* Δ*gtfM*^+^, *T. forsythia* Δ*gtfI*^+^, *T. forsythia* Δ*gtfL*^+^, and *T. forsythia* Δ*gtfE*^+^) confirmed the synthesis of the native decasaccharide (**Supplementary Figure S9**). Data for *T. forsythia* Δ*gtfS*^+^ were published previously (Tomek et al., 2017).

To obtain insight into the specificity of the Gtfs GtfSMILE in the biosynthesis of the *T. forsythia O-*glycan, ESI-MS analysis of β-eliminated *O-*glycans from TfsB (**Figure 3C**) and TfsA (data not shown) as present in the Δ*gtfS*, Δ*gtfM*, Δ*gtfI*, Δ*gtfL*, and Δ*gtfE* mutants was performed. The doubly charged wild-type decasaccharide exhibited an *m*/*z* = 949.9 [M+2H]^2+^, which corresponds to *m*/*z* = 1898.8 when calculating the singly charged form thereof, confirming the composition of the previously elucidated *O-*glycan structure (Posch et al., 2011) (**Figure 3C**, i). Structure-wise, the position of the branching Fuc residue that has remained ambiguous from our initial investigations could now be determined, using a Δ*gtfL* mutant (Δ*Tanf_01300*) (see below).

In comparison to the wild-type, the signal for the largest glycan produced by a Δ*gtfS* (Δ*Tanf_01245*) mutant was detected at *m*/*z* = 1523.4 [M+H]^+^, which agrees with the *m*/*z* value of the *T. forsythia O-*glycan lacking the terminal Pse5Am7Gra residue (361.2 Da) and one methyl group (14.0 Da). A detailed analysis of the GtfS protein was performed recently in our group and revealed that it has α-2,4 Pse5Am7Gra transferase activity (Tomek et al., 2017) (**Figure 3C**, ii). The Δ*gtfM* mutant (Δ*Tanf_01260*) lacks the 4-*O-*methyl-*N-*acetylmannosaminuronic acid residue (231.1 Da) in addition to Pse5Am7Gra, making the signal at *m*/*z* = 1306.4 [M+H]^+^ the largest detectable glycan for this mutant (**Figure 3C**, iii). The MS data together with the *T. forsythia* ATCC 43037 *O-*glycan structure (compare with **Figure 1A**) are, therefore, indicative of GtfM having a β-1,3-*N*-acetylmannosaminuronic acid transferase activity. The glycan is further truncated upon knocking-out of *gtfI* (Δ*Tanf_01290*). In this mutant glycan, the three-sugar branch composed of the Pse5Am7Gra, an *N-*acetyl mannosaminuronic acid and the 4-*O-*methyl-*N*-acetyl mannosaminuronic acid residue (in total 809.1 Da), is absent and results in a signal corresponding to the ammonium adduct ion at *m*/*z* = 1106.4 [M+NH_4_]^+^ (**Figure 3C**, iv). Thus, the proposed enzymatic function of GtfI is that of a β-1,4-*N-*acetylmannosaminuronic acid transferase.

**FIGURE 4:**
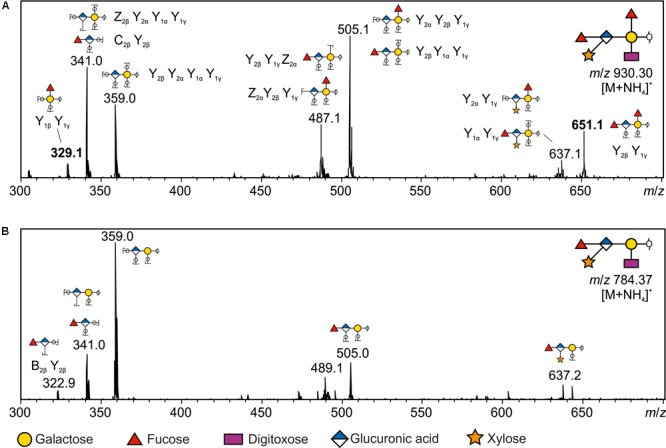
Positive mode CID tandem spectra of reduced *O*-glycans from glycoproteins in *T. forsythia*Δ*Tanf_01300*. **(A)** The *m*/*z* = 930.3^1+^ precursor ion corresponds to the glycan composition Gal + GlcA + Xyl + Dig + Fuc_2_ containing an ammonium adduct [M+NH_4_]^+^. Its product ion spectra contained several typical Y/Z and a few B/C ion cleavages that were highly informative to sequence the proposed glycan structure. For example, the C_2β_Y_2β_ (*m*/*z* = 341) and B_2β_Y_2β_ (*m*/*z* = 322.9; **B**) ions clearly show that the GlcA is linked to a Fuc at the non-reducing end. Similarly, the reducing end Y_1β_Y_1γ_ double cleavage ion of *m*/*z* = 329 is indicative of a Fuc residue linked to the reducing-end Gal. Furthermore, this product ion spectra allows to clearly distinguish the presence of the additional fucose residue, which is lacking in the product ion spectra of *m*/*z* 784.37^1+^ **(B)**. Unique fragment ions (*m*/*z* = 329 and 651; bold) additionally supporting the location of the additional Fuc residue are only observed in the product ion spectra of *m*/*z* = 930.3^1+^. **(B)** Product ion spectra from [M+NH_4_]^+^ ions of *m*/*z* = 784.37 corresponding to the composition Gal + GlcA + Xyl + Dig + Fuc. Monosaccharide symbols are according to the Symbol Nomenclature for Glycans (SNFG) (Varki et al., 2015).

The *m*/*z* = 930.3 [M+NH_4_]^+^ signal of a Δ*gtfL* mutant (Δ*Tanf_01300*) could initially not be assigned to the published *O*-glycan structure (Posch et al., 2011). In our previous study, a fragment ion of *m/z* = 341.0 was tentatively interpreted as the single cleavage C-ion Fuc-MeGal. However, the *m*/*z* = 930.3 glycan of the Δ*gtfL* mutant, which contains two fucoses and lacks the distal Gal, necessitated another interpretation of this fragment (**Figure 4**), leading to a reconsideration of the location of the second fucose residue. Inspection of the fragment spectrum of *m*/*z* = 930.3^1+^ revealed an *m*/*z* = 329.0 fragment not present in the monofucosylated *m*/*z* = 784.6^1+^ peak (**Figure 4**). This Y_1β_Y_1γ_ double cleavage ion strongly indicated that a Fuc residue is attached to the reducing-end Gal. This interpretation was supported by the fragment ion *m*/*z* = 651.1, which was observed in the product ion spectra of *m*/*z* = 930.3^1+^. Thus, these findings prove that the glycan of this mutant is composed of the six innermost sugar residues, and that GtfL acts as an α-1,2 galactosyltransferase (**Figures 1A, 3C**, v). Simultaneously, β-1,4 *N-*acetylmannosaminuronic acid transfer activity of GtfI seems to be affected by the deletion of GtfL, since the Pse5Am7Gra-containing trisaccharide branch is also missing in this structure, indicating the requirement of either a distinct composition of the acceptor for recognition by the enzyme or the necessity of association of GtfI with GtfL for enzymatic activity. Finally, fucosyltransferase activity of GtfE is supported by the analysis of a Δ*gtfE* knock-out mutant (Δ*Tanf_01305*) which reveals an ammonium adduct mass of *m*/*z* = 784.2 [M+NH_4_]^+^ that represents an *O-*glycan composed of the five innermost sugar residues (pentasaccharide core) (**Figure 3C**, vi).

These data are complemented by previous results from our group showing that the *O-*glycan from a *wecC* knock-out mutant (Δ*Tanf_01280*) lacks the same three-sugar branch as the Δ*gtfI* mutant (Posch et al., 2011). We concluded that WecC fulfills its predicted role as a UDP-*N-*acetylmannosaminuronic acid dehydrogenase which works in concert with the predicted UDP-*N-*acetylglucosamine 2-epimerase WecB (Tanf_01285) to synthesize UDP-*N-*acetylmannosaminuronic acid prior to its transfer to the branching Fuc residue of the *T. forsythia O-*glycan structure (compare with **Figure 1A**), catalyzed by GtfI (**Figure 3C**, iv).

Summarizing, the *T. forsythia* protein *O*-glycosylation gene cluster encodes all necessary information for the assembly of the species-specific portion of the decasaccharide.

### Three Methyltransferases Modify the *O*-Glycan With Two Methyl Groups

**FIGURE 5:**
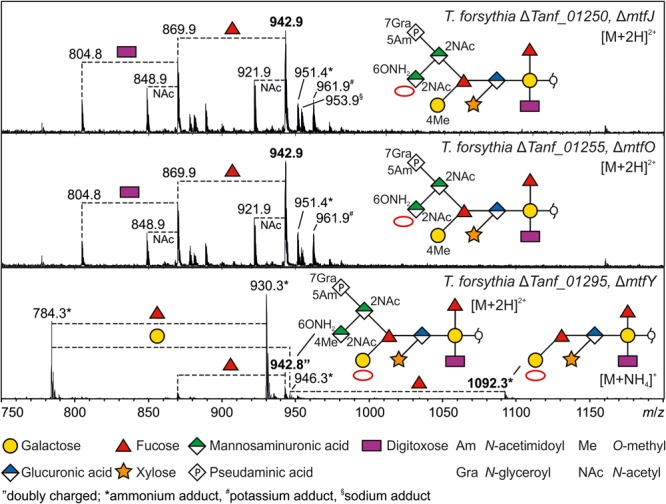
ESI-MS sum spectra of β-eliminated TfsB *O*-glycans from *T. forsythia* ATCC 43037 methyltransferase knock-out mutants. The glycan structures of the signals corresponding to the largest mass (bold *m*/*z* values) are shown in SNFG representation (Varki et al., 2015). Other *O*-glycan signals detected for the respective mutants were assigned based on the *m*/*z* mass differences corresponding to the loss of individual sugar units and/or modifications. The lack of methyl modifications is indicated by a red circle in the symbolic *O*-glycan structure representation.

While two methylated sugars are present in the *T. forsythia* ATCC 43037 *O-*glycan structure, three putative Mtfs are encoded within the protein *O-*glycosylation gene cluster (MtfJOY). To test the methylation activity and specificity of these predicted enzymes, single gene knock-outs were constructed (**Supplementary Figures S7–S9**). *O*-Glycans of mutants, deficient in either the Mtf gene *mtfJ* (Δ*Tanf_01250*) or *mtfO* (Δ*Tanf_01255*), experienced a loss of the 4-*O-*methyl group at the terminal *N-*acetyl mannosaminuronamide, as MS analyses revealed (**Figure 5**; red circles indicate the absence of the methyl groups). The expected loss of 14 Da resulted in a doubly charged ion at *m*/*z* = 942.9 [M+2H]^2+^ as detected for the analyzed TfsB (**Figure 5**) and TfsA (data not shown) *O-*glycans of either Mtf mutant. While MtfJ and MtfO obviously catalyze methyl transfer to the same sugar residue, these proteins differ in length and do not share significant sequence similarity. In contrast, the *O-*glycan of the Δ*mtfY* mutant (Δ*Tanf_01295*) lacked the 4-*O-*methyl group on the distal Gal residue (**Figure 5**). The discrimination which of the two methyl groups was affected in the different Mtf mutants was inferred from the occurrence of unique glycan fragments obtained during MS and MS/MS analyses (data not shown).

### Protein Glycosylation Affects Immunogenicity of *T. forsythia*

We proceeded to investigate whether differential cell surface protein *O-*glycosylation as present in the Gtf-deficient mutants of *T. forsythia* ATCC 43037 impacts the bacterium’s overall immunogenicity. For this endeavor, we selected two mutants with distinct *O-*glycan compositions for which we expected differential responses of infected immune cells in comparison to the wild-type. This included Δ*gtfS*, lacking the sialic acid mimic Pse5Am7Gra (Tomek et al., 2017) and Δ*gtfE* (this study) producing the pentasaccharide core only and, thus, exposing a terminal Fuc residue. Further, Δ*gftI* which produces the same truncated glycan as Δ*wecC* (Δ*Tanf_01280*) (Posch et al., 2011) was included as a control, since for that mutant reference immunology data is available in the literature (Settem et al., 2013).

Dendritic cells as central communicators linking innate and adaptive immune responses possess a wide variety of PRRs that allow them to recognize and quickly respond to the presence of opportunistic pathogens (Reis e Sousa, 2006; Merad et al., 2013). The high selectivity of these PRRs enables DCs to fine-tune the outcome of the immune response, depending on the molecular characteristics of the stimulus (Lutz and Schuler, 2002). Therefore, BMDCs and human monocyte-derived DCs were used to explore how the loss of certain sugar residues of the *T. forsythia O*-glycan affects activation and release of inflammatory cytokines by these cells as well as the subsequent polarization of an adaptive T cell response.

**FIGURE 6:**
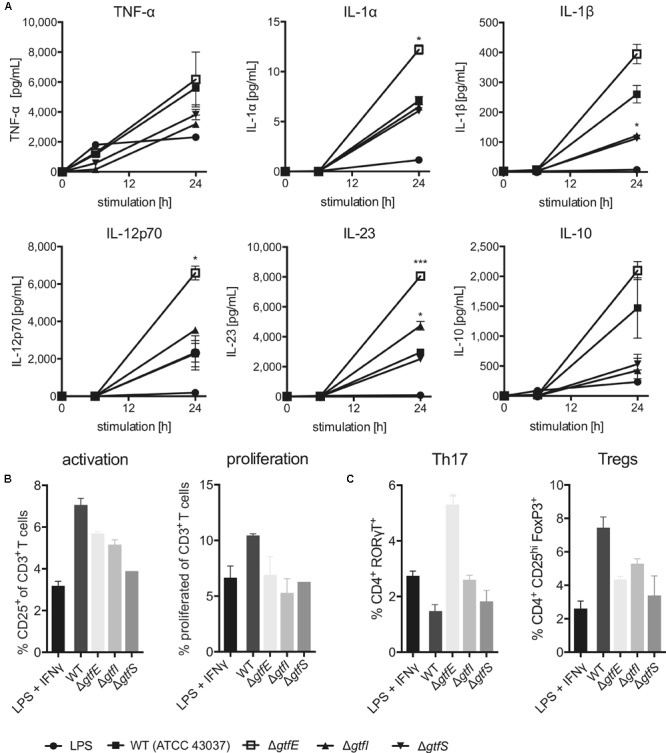
Effects of protein *O*-glycosylation on *T. forsythia* immunogenicity. **(A)** Secretion of inflammatory cytokines by human DCs upon stimulation with *T. forsythia* wild-type (WT) and glycosyltransferase-deficient mutants (*T. forsythia* Δ*gtfE*, Δ*gtfI*, and Δ*gtfS*) as measured in culture supernatants by ProcartaPlex Multiplex Immunoassay. **(B,C)** T cell-priming upon APC stimulation with *T. forsythia* wild-type and glycosyltransferase-deficient mutants was assessed by culturing PBMCs. **(B)** T cell activation was measured via expression of CD25 by flow cytometry. Cells were pre-gated for live CD3^+^ cells, T cell proliferation was assessed after 8 days by CFSE dilution,. **(C)** CD4^+^ T cell differentiation was assessed by expression of signature transcription factors for Th17 (RORγT) and Treg (FoxP3) cells as measured by flow cytometry. All data are presented as mean ± SEM of triplicate determinations. One representative out of three **(A)** and two **(B,C)**, respectively, independent experiments is shown. Statistically significant differences in **(A)** are indicated as ^∗^*p* < 0.05 and ^∗∗∗^*p* < 0.001 (Student’s *t*-test). LPS, *E. coli* O111:B4 LPS.

First, the surface expression of DC maturation markers, including MHC-II, CD80 and CD86, upon stimulation by *T. forsythia* wild-type and the Δ*gtfS*, Δ*gtfI*, and Δ*gtfE* mutants was investigated. Over a period of 24 h, all of these activation markers were up-regulated in response to stimulation and did not reveal any differences between the tested strains (**Supplementary Figure S10A**). As this suggested no effect on the overall activation of DCs, their functionality was tested next. While secretion of most pro-inflammatory cytokines was not altered between the wild-type and the Gtf-deficient mutants, Δ*gftE* resulted in a profound increase in IL-1β, IL-12, and IL-23 release by human monocyte-derived DCs over 24 h post-stimulation (**Figures 6A** and **Supplementary Figure S10B**), and in TNF-α and IL-6, as observed with mouse-derived DCs (**Supplementary Figure S11**). Striking differences were observed with regard to IL-1β production; while Δ*gftE* enhanced secretion of this key inflammatory cytokine in human DCs, Δ*gftI* and Δ*gftS* yielded a significant decrease when compared to the wild-type (**Figure 6A**). A similar effect was observed for IL-10 (**Figure 6A**), which acts as an important immunosuppressive mediator and is required to dampen ongoing inflammatory responses.

These results prompted us to explore the T cell-priming potential of antigen-presenting cells (APCs) upon stimulation by different *T. forsythia* glycosylation mutants. We therefore cultured PBMCs, containing APCs and T cells, for 8 days in the presence of different *T. forsythia* strains. With regard to both activation (CD25 up-regulation) and proliferation, a decline in T cell activation upon stimulation with all Gtf-deficient mutants as compared to the wild-type could be observed (**Figure 6B**). However, dissecting the polarization of the resulting T cell response, we observed a remarkable induction of RORγT-expressing CD4^+^ T cells by Δ*gftE*-stimulated DCs, whereas only a minor effect was seen for the other tested strains (**Figure 6C**). This suggests a trend toward enhanced differentiation of Th17 cells in response to *T. forsythia* glycan truncation, an effect that has been previously reported to be of relevance for the progression of periodontitis (Settem et al., 2013). Critically, this observation is reinforced by the profound increase in IL-23 and IL-1β we found upon human DC stimulation with Δ*gftE* (**Figure 6A**), as these cytokines represent key mediators of Th17 polarization (Iwakura and Ishigame, 2006; Sutton et al., 2009). The differentiation of Tregs was similarly reduced by all truncated glycans in comparison to the wild-type (**Figure 6C**), indicating an overall involvement of the *O*-glycan in maintaining a suppressive T cell environment. Moreover, a minor trend toward decreased induction of Th1 (IFN-γ^+^) cells could be observed (**Supplementary Figure S10C**), which corresponds to a slightly reduced IFN-γ secretion by human DCs stimulated with *T. forsythia* Gtf-deficient mutants (**Supplementary Figure S10B**).

## Discussion

Periodontitis is an inflammatory disease that is highly prevalent among the adult population world-wide. It involves the periodontal pathogens *T. forsythia, Porphyromonas gingivalis*, and *Treponema denticola*, together constituting the so-called “red complex” of bacteria (Kassebaum et al., 2014; Hajishengallis, 2015). In *T. forsythia*, the prominent and unique S-layer glycoproteins TfsA and TfsB are virulence factors (Sharma, 2010). While previous studies were designed to elucidate the S-layer *O*-glycan structure (Posch et al., 2011; Friedrich et al., 2017), the S-layer ultrastructure (Sekot et al., 2012; Oh et al., 2013), and the immunological properties of the S-layer glycoproteins (Yoneda et al., 2003; Sakakibara et al., 2007; Sekot et al., 2011; Chinthamani et al., 2017), only few data is available on the impact of *T. forsythia*’s cell surface glycosylation on the modulation of host immunity (Settem et al., 2013, 2014). This is surprising given the fact that *T. forsythia O-*glycans are highly abundant on the bacterial cell surface due to their display via the S-layer matrix (Sekot et al., 2012), making them prone to act at the bacterium-immune interface.

To provide a basis for validating *T. forsythia*’s S-layer protein *O*-glycosylation as a possible target against periodontitis, within the frame of this study (i) the prevalence of an underlying protein *O-*glycosylation gene cluster in the genomes of different *T. forsythia* strains was assessed, (ii) insights into the *O-*glycan biosynthesis pathway were obtained, and (iii) a relationship between *O*-glycan structure and immunogenicity was delineated.

In this study, the general protein *O-*glycosylation gene cluster of *T. forsythia* ATCC 43037 was extended to span 27-kb on the bacterium’s genome (Friedrich et al., 2015). For 18 out of the 26 ORFs, carbohydrate metabolism-related functions were predicted. These include, a Wzx-like flippase for translocation of the glycan moiety to the periplasmic space prior to protein transfer, six enzymes needed for the synthesis of CMP-Pse (PseBCFHGI) as well as two enzymes for the synthesis of UDP-linked *N-*acetyl mannosaminuronic acid (WecB, WecC), five Gtfs (GtfSMILE), three Mtfs (MtfJOY), and one ORF putatively involved in the Am-modification of the Pse residue (**Figure 1B**).

Genome comparisons of *T. forsythia* strains revealed that all seven strains included in this study have the genetic potential for protein *O*-glycosylation (**Figure 2**). A high degree of conservation of the protein *O-*glycosylation gene clusters with regard to content and organization of genes became evident, with a possible grouping of strains according to the type of terminal nonulosonic acid synthesized (Pse *versus* Leg), with only one strain presenting an ambiguous situation. As a common feature, the *O*-glycosylation gene clusters in all analyzed strains start with orthologs of the Gtf genes *gtfE* (*Tanf_01305*) and *gtfL* (*Tanf_01300*) and terminate with an ortholog of the flippase gene *wzx* (*Tanf_01180*), which is identical to the situation found in 24 other *Bacteroides* species (Coyne et al., 2013). While orthologs of *wzx* and *gtfE* are also predicted in the genome of the periodontal health-associated isolate *Tannerella* sp. HOT-286 (Beall et al., 2014), none of the intervening genes related to carbohydrate metabolism as present in the pathogenic strains are confidently predicted. Thus, it is conceivable that the potential of complex protein *O-*glycosylation is a specific trait of periodontitis-associated *T. forsythia* strains, especially when considering that cell surface glycosylation in bacteria can modulate immune responses during pathogenesis (Szymanski and Wren, 2005; Lebeer et al., 2010; Valguarnera et al., 2016).

**FIGURE 7:**
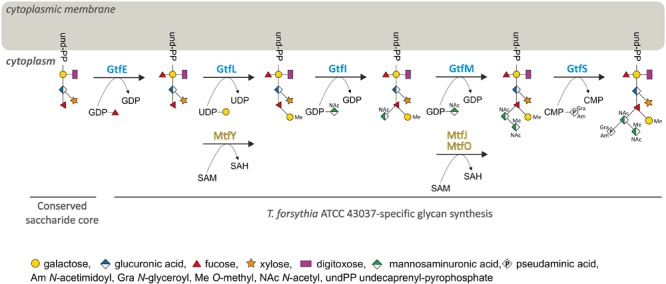
Model for the biosynthesis of the species-specific portion of the *T. forsythia* ATCC 43037 *O*-glycan. Upon synthesis of the pentasaccharide core on an undP lipid carrier, the first carbohydrate residue of the species-specific glycan is a Fuc residue conferred by GtfE. The glycan is elongated with a Gal residue which is transferred by GtfL and methylated by MtfY. The assembly of the three sugar branch, consisting of a ManNAcA residue (transferred by GtfI), a ManNAcCONH_2_ residue (GtfM), which is methylated by either MtfJ or MtfO, and a Pse5Am7Gra residue (transferred via GtfS), completes the synthesis of the decasaccharide.

Experimentally, we have determined the roles of five Gtfs and three Mtfs encoded in the protein *O-*glycosylation gene cluster by analysing the protein-released glycans of the respective knock-out mutants. We could establish an assembly line for the *T. forsythia*-specific portion of the *O-*glycan by subsequent involvement of the Gtfs GtfELIMS (**Figure 7**). While the underlying genetic information is encoded in the gene cluster, the information for the assembly of the pentasaccharide core would be encoded elsewhere on the bacterial genome. A putative *O-*OTase transferring the glycan onto the acceptor proteins could not be identified for *T. forsythia*, so far. This leaves the question open of whether this *O-*glycan is synthesized by an OTase-dependent or OTase-independent mechanism (Choi et al., 2010; Grass et al., 2010; Schwarz et al., 2011; Iwashkiw et al., 2013). Coyne et al. (2013) suggested two possible models for protein *O*-glycosylation in *Bacteroidetes*, exemplified with *B. fragilis*. According to the first model, the glycan core and the species-specific glycan would be built separately on an undecaprenyl-phosphate (undP) lipid carrier and flipped to the periplasmic side of the cytoplasmic membrane by individual Wzx flippases. The core glycan would be linked to the protein by an as yet unidentified OTase, followed by linking of the species-specific glycan portion to the core by an *O*-antigen ligase-like protein. Alternatively, the species-specific glycan might be added to the core in the cytoplasm and then a single flippase would transfer the entire glycan to the periplasm for addition to proteins (Fletcher et al., 2009; Coyne et al., 2013). Considering our experimental set-up we cannot make any conclusion as to which of the two models would be valid for *T. forsythia*.

Concerning 4-*O-*methylation of the distal Gal and the *N*-acetyl mannosaminuronamide residue, there is redundancy of enzymes, since the latter residue can be methylated by both MtfJ and MtfO, while MtfY recognizes only the distal Gal residue as a substrate. It remains to be investigated if MtfJ and MtfO might be differentially expressed in *T. forsythia* as a function of growth conditions or other signals and, generally, if methylation occurs at the nucleotide-sugar level or after sugar transfer to the nascent glycan. Methylation of bacterial glycans is a rarely reported event. Although methylation of the non-reducing-end sugar of a nascent polysaccharide chain has been discussed as a stop-signal for glycan chain elongation (Clarke et al., 2011), a clear determination of its biological function is still missing, especially when occurring at multiple sites of the glycan (Staudacher, 2012). *O*-Methylated glycans were recently suggested to constitute a conserved target of the fungal and animal innate immune system (Wohlschlager et al., 2014). All three Mtf-deficient *T. forsythia* mutants from our study might aid in defining the roles of *O-*methylated sugars in a bacterial context.

In the natural habitat of the oral cavity, *T. forsythia* finds itself in an area of conflict between the requirement of inflammation to procure nutrients from tissue breakdown and the necessity to evade immune-mediated killing (Settem et al., 2013, 2014). Similar to other inflammatory diseases, disruption of the proper balance between individual subtypes of Th cells contributes to the progression of periodontitis (Baker, 2000). In a previous study using a *T. forsythia* Δ*wecC* glycosylation mutant (representing the same glycan structure as the Δ*gtfI* mutant of the current study), it was found that truncation of the *O*-glycan translated into a robust Th17 response with the consequence of reduced alveolar bone loss in mice. Additionally, the Δ*wecC* mutant was increasingly susceptible to neutrophil-mediated clearance. Based on these data a fundamental role of *T. forsythia* wild-type cell surface glycosylation in restraining the Th17 response and ensuring the persistence of the pathogen in the host was proposed (Settem et al., 2013). The attenuated, Th17-biasing glycosylation mutant devoid of the *O-*glycan’s trisaccharide branch was effective in blocking *P. gingivalis* persistence in a periodontitis mouse model (Settem et al., 2014), unraveling the *T. forsythia O-*glycan as a means for influencing the pathogenesis of periodontitis. However, it is still under debate if Th17 responses have protective or destructive roles in inflammatory diseases, since this might depend on the specificity and phase of the disease (Ohyama et al., 2009).

Using *T. forsythia* mutants with defined, truncated *O*-glycans, we attempted to obtain clearer insight into the *O-*glycan structure–function relationship at the immune interface. Analysis of DC maturation markers did not reveal differential activation by the glycosylation mutants in comparison to the wild-type. In terms of release of proinflammatory cytokines by human DCs, only *T. forsythia*Δ*gftE* resulted in a profound increase in TNF-α, IL-6, IL-1β, IL-12, and IL-23 release over 24 h post-stimulation (**Figures 6A** and **Supplementary Figure S11**). Especially with the enhanced secretion of IL-1β and IL-23, which are key mediators of Th17 polarization (Iwakura and Ishigame, 2006; Sutton et al., 2009), this mutant showed a striking difference to Δ*gftI* and Δ*gftS* where a decrease of these cytokines was found (**Figure 6A**). In line with these data, also the analysis of the T cell-priming potential of APCs upon stimulation revealed *T. forsythia*Δ*gftE* as a unique stimulus. Here, a remarkable induction of RORγT-expressing CD4^+^ T cells was observed, whereas only a minor effect was seen for the other tested mutants (**Figure 6C**). This suggests an enhanced differentiation of Th17 cells in response to *T. forsythia* glycan truncation, which was most pronounced upon stimulation with the pentasaccharide core only. For the Δ*gftI* mutant, equaling *O*-glycan structure-wise the Δ*wecC* mutant for which this effect had been reported before (Settem et al., 2013), a comparably minor effect was seen. We conclude that a more truncated version of the *O-*glycan is even more favorable for inducing a robust Th17 response. *Vice versa*, Th17 suppression is maintained regardless of the presence of the residues *N*-acetyl mannosaminuronic acid, *N*-acetyl mannosaminuronamide and Pse5Am7Gra. This implicates that the phylum-wide conserved core as present in Δ*gftE* is responsible for modulating DC effector functions by Th17 activation, while the species-specific glycan portion, either in complete or truncated form, is required for maintaining a Th17 suppressive environment. This might contribute to *T. forsythia*’s strategy as part of a dysbiotic microbial community to resist immune elimination and create permissive conditions for growth in a nutritionally favorable environment.

## Author Contributions

CS and MT conceived the study, designed the experiments, developed the methodology, and wrote the manuscript. MT, DM, MW, VF, BJ, KF, LN, IN, NZ, AE-D, and DK performed the experiments. MT, DM, NZ, JD, AE-D, DK, HH, FA, and CS analyzed the data. All authors read and approved the submitted version of the manuscript. Written informed consent was obtained from all authors of the study.

## Conflict of Interest Statement

The authors declare that the research was conducted in the absence of any commercial or financial relationships that could be construed as a potential conflict of interest.

## Footnotes

1 https://blast.ncbi.nlm.nih.gov

2 http://pfam.xfam.org

3 ftp://ftp.ncbi.nlm.nih.gov/genomes

## Abbreviations

Ac: *N*-acetyl or acetamido
Am: *N*-acetimidoyl or acetamidino
APC: antigen presenting cell
BHI: brain heart infusion
BMDC: bone marrow-derived dendritic cell
Cat: chloramphenicol
cDNA: copy DNA
CFSE: carboxyfluorescein succinimidyl ester
CMP: cytidine-5′-monophosphate
Dig: digitoxose
DC: dendritic cell
DMEM: Dulbecco’s Modified Eagle’s Medium
Erm: erythromycin
ESI: electrospray ionization
Fuc: fucose
Gal: galactose
Gc: *N*-glycolyl
GDP: guanosine-5′-diphosphate
GM-CSF: granulocyte-macrophage colony-stimulating factor
Gra: *N*-glyceroyl or *N*-2,3-dihydroxypropionyl or glycerate group
Gtf: glycosyltransferase
IL: interleukin
INF: interferon
LB: lysogeny broth
LC: liquid chromatography
Leg: legionaminic acid (Leg5,7Ac_2_), 5,7-diacetamido-3,5,7,9-tetradeoxy-D-*glycero*-D-*galacto*-nonulosonic acid
LFG: locus for glycosylation
LPS: lipopolysaccharide
MHC: major histocompatibility complex
Mtf: methyltransferase
MS: mass spectrometry
OP: Octaplas medium
ORF: open reading frame
OTase: oligosaccharyltransferase
PBMC: peripheral blood mononuclear cell
PGC: porous graphitized carbon
PRR: pattern recognition receptor
Pse: pseudaminic acid (Pse5,7Ac_2_), 5,7-diacetamido-3,5,7,9-tetradeoxy-L-*glycero*-L-*manno*-nonulosonic acid
SAM: *S*-adenosyl-L-methionine
SDS-PAGE: sodium dodecyl sulfate-polyacrylamide gel electrophoresis
Th: T helper cell
TNF: tumor necrosis factor
Tregs: regulatory T cells
UDP: uridine-5′-diphosphate
und-PP: undecaprenyl pyrophosphate
Xyl: xylose
