# Supplementary material for: A General Protein O-Glycosylation Gene Cluster Encodes the Species-Specific Glycan of the Oral Pathogen Tannerella forsythia: O-Glycan Biosynthesis and Immunological Implications
Source: Front Microbiol. 2018 Aug 28;9:2008. doi: 10.3389/fmicb.2018.02008 (PMC6120980; doi:10.3389/fmicb.2018.02008)
Supplement: Supplementary file 1 [file Data_Sheet_1.pdf]

Supplementary Material

A General Protein *O*-Glycosylation Gene Cluster Encodes the Species-Specific Glycan of the Oral Pathogen *Tannerella forsythia*: Glycan Biosynthesis and Immunological Implications

Markus B. Tomek, Daniel Maresch, Markus Windwarder, Valentin Friedrich, Bettina Janesch, Kristina Fuchs[1], Laura Neumann, Irene Nimeth, Nikolaus F. Zwickl, Juliane C. Dohm, Arun Everest-Dass, Daniel Kolarich, Heinz Himmelbauer, Friedrich Altmann, and Christina Schäffer[*]

*** Correspondence:** Christina Schäffer, christina.schaeffer@boku.ac.at

Supplementary Data

Supplementary Figures S1-S11

Supplementary Tables S1-S5

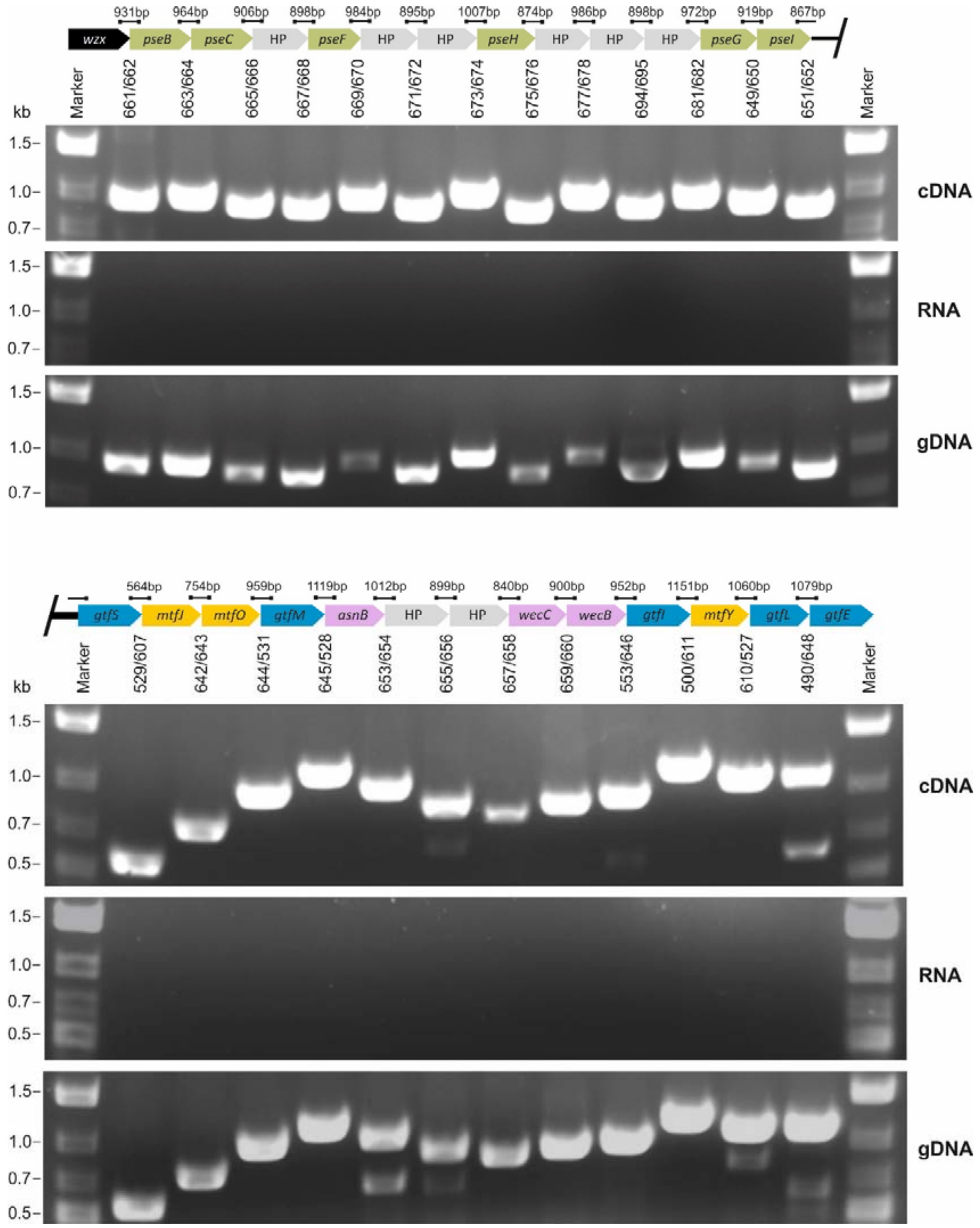

Supplementary FIGURE S1 │ Operon structure of the *T. forsythia* ATCC 43037 protein *O*-glycosylation gene cluster. Agarose gel electrophoresis analyses of co-transcription of neighbouring genes as indicated, based on expected PCR fragment sizes upon PCR amplification from cDNA (upper panel), total RNA (middle panel; negative control) and genomic DNA (lower panel; positive control). Primers are listed in Supplementary Table S2. The presence of PCR fragments in the cDNA gels demonstrates co-transcription of all analysed genes, indicative of a polycistronic *O*-glycosylation gene cluster. O´Gene Ruler 1 kb Plus DNA Ladder (Thermo Fisher Scientific) was used as a gene ladder and is indicated on the first and last lane.

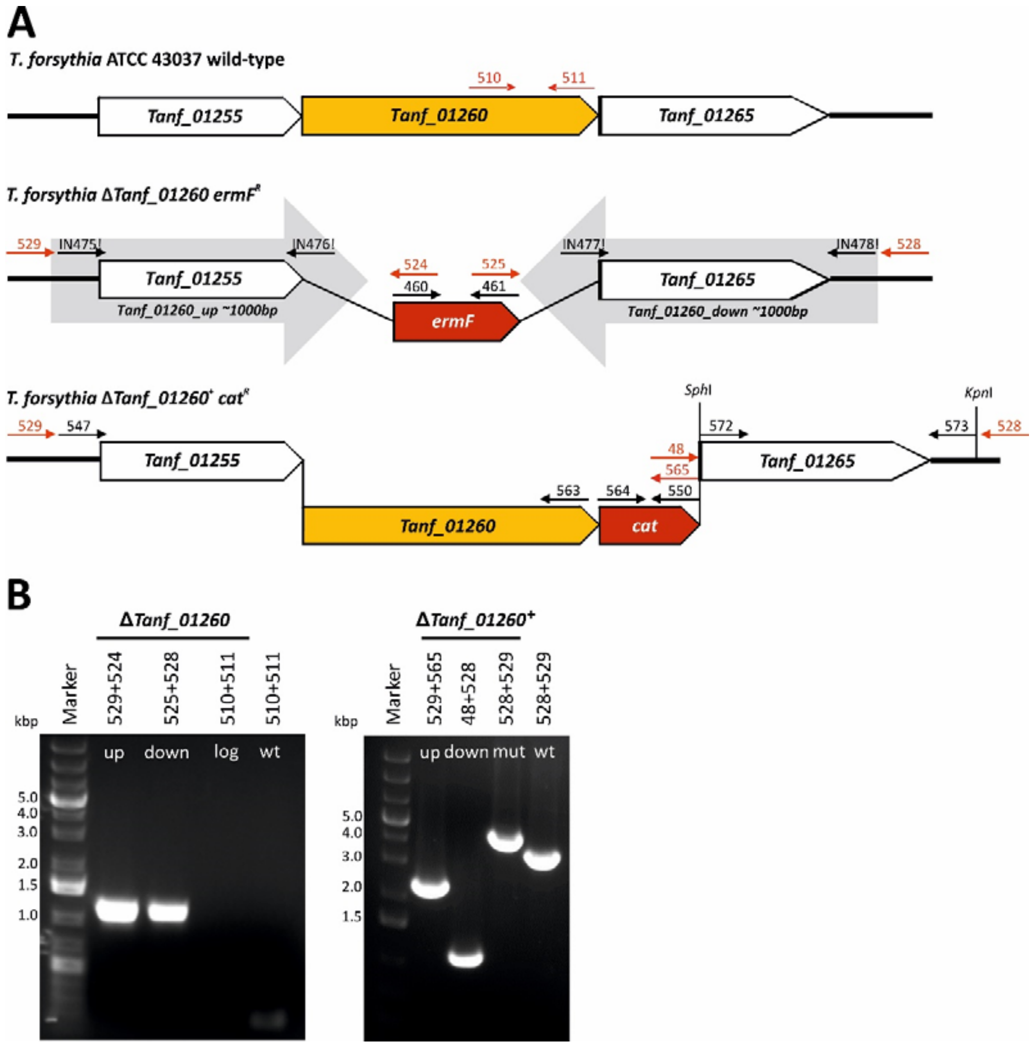

Supplementary FIGURE S2 │ Strategy for the generation of a *T. forsythia* ATCC 43037 mutant at the *Tanf_01260* locus and confirmation by PCR. (A) The genomic organization of the *Tanf_01260* locus is shown for the parent strain *T. forsythia* ATCC 43037, the Δ*Tanf_01260* mutant and the reconstituted mutant Δ*Tanf_01260*$^{+}$. Black coloured arrows represent primers used for PCR amplification of genes and homologous regions, red coloured primers represent those used to screen for correct integration of the knock-out and reconstitution cassettes; restriction sites used for cloning are indicated (not drawn to scale). **(B)** Agarose gel electrophoresis (left) confirms the deletion of *Tanf_01260* using the upstream primers 529/524 (1099 bp) and downstream primers 525/528 (1089 bp) on genomic DNA of *T. forsythia* ATCC 43037 Δ*Tanf_01260* mutant with integrated *ermF* cassette. Primers 510/511 yield in a 199-bp PCR fragment when using *T. forsythia* wild-type genomic DNA, whereas this fragment is absent on genomic DNA of the Δ*Tanf_01260* mutant confirming the loss of the gene (log). Agarose gel electrophoresis (right) confirmed the reconstitution of the deleted *Tanf_01260* gene using upstream primers 529/565 (2247 bp) and downstream primers 48/528 (1095 bp). Screening primers 528/529 yield a 3937-bp PCR product on genomic DNA of the reconstituted mutant Δ*Tanf_01260*$^{+}$ (mut) with integrated *cat* resistance gene, whereas the same primer pair results in a 3280-bp product on genomic DNA of the *T. forsythia* wild-type. O´Gene Ruler 1 kb Plus DNA Ladder (Thermo Fisher Scientific) was used as a gene ladder.

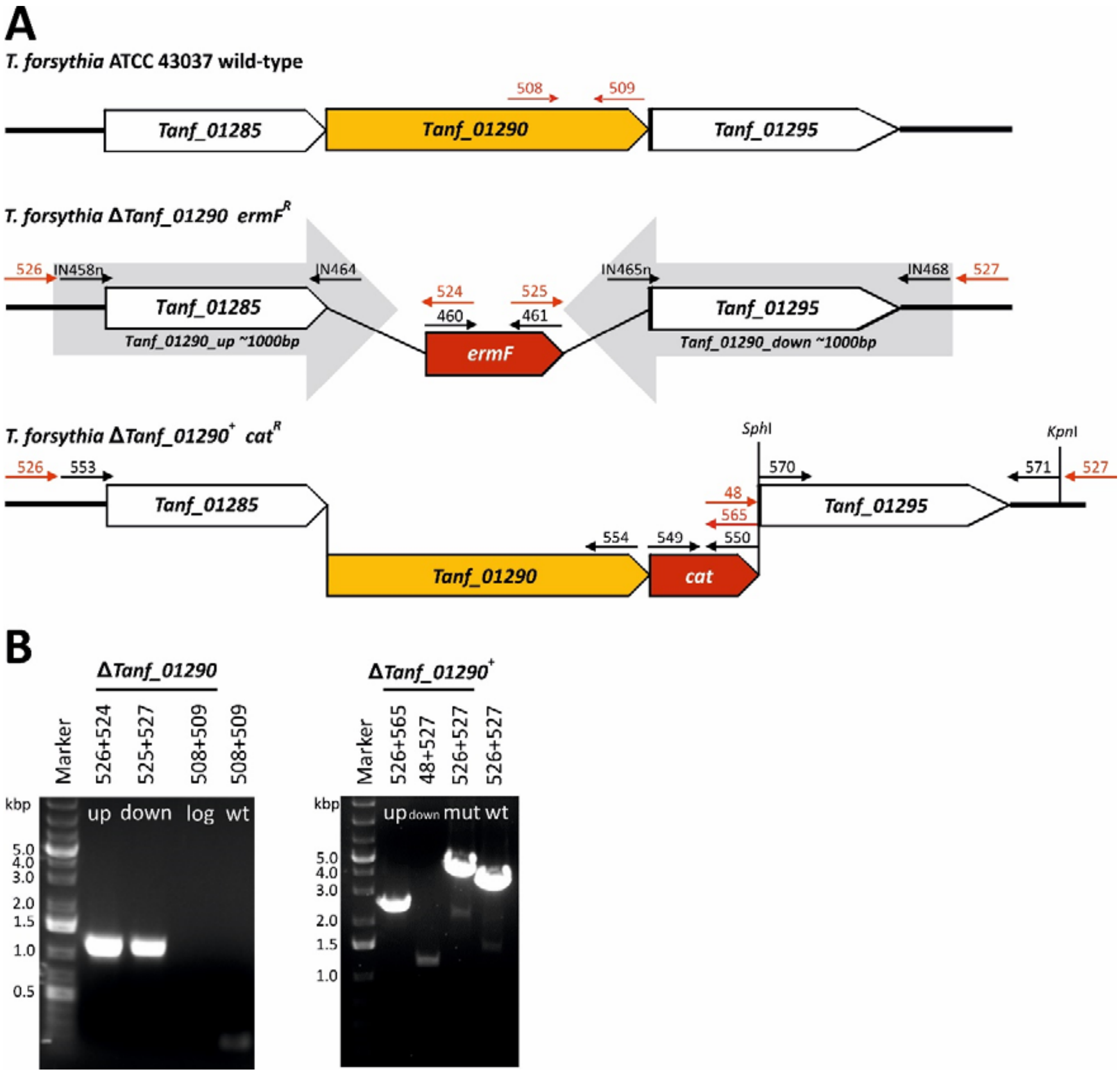

Supplementary FIGURE S3 | Strategy for the generation of a *T. forsythia* ATCC 43037 mutant at the *Tanf_01290* locus and confirmation by PCR. (A) The genomic organization of the *Tanf_01290* locus is shown for the parent strain *T. forsythia* ATCC 43037, the Δ*Tanf_01290* mutant and the reconstituted mutant Δ*Tanf_01290*$^{+}$. Black coloured arrows represent primers used for PCR amplification of genes and homologous regions, red coloured primers represent those used to screen for correct integration of the knock-out and reconstitution cassettes; restriction sites used for cloning are indicated (not drawn to scale). **(B)** Agarose gel electrophoresis (left) confirms the deletion of *Tanf_01290* using the upstream primers 526/524 (1150 bp) and downstream primers 525/527 (1159 bp) on genomic DNA of *T. forsythia* ATCC 43037 Δ*Tanf_01290* mutant with integrated *ermF* cassette. Primers 508/509 yield in a 193-bp PCR fragment when using *T. forsythia* wild-type genomic DNA, whereas this fragment is absent on genomic DNA of the Δ*Tanf_01290* mutant confirming the loss of the gene (log). Agarose gel electrophoresis (right) confirms the reconstitution of the deleted *Tanf_01290* gene using upstream primers 526/565 (2358 bp) and downstream primers 48/527 (1156 bp). Screening primers 526/527 yield a 4118-bp PCR product on genomic DNA of the reconstituted mutant Δ*Tanf_01290*$^{+}$ (mut) with integrated *cat* resistance gene, whereas the same primer pair results in a 3461-bp product on genomic DNA of the *T. forsythia* wild-type. O´Gene Ruler 1 kb Plus DNA Ladder (Thermo Fisher Scientific) was used as a gene ladder.

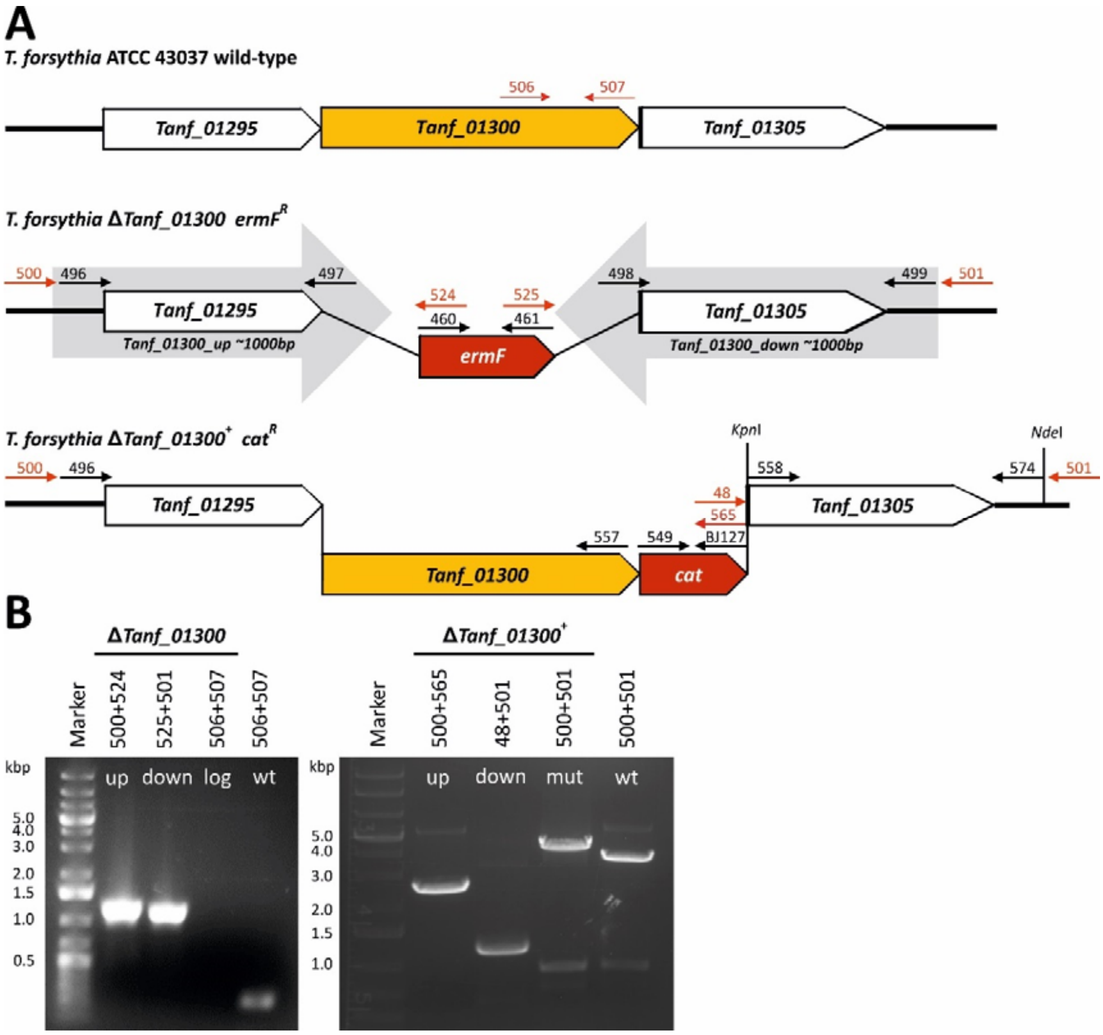

Supplementary FIGURE S4 | Strategy for the generation of a *T. forsythia* ATCC 43037 mutant at the *Tanf_01300* locus and confirmation by PCR. **(A)** The genomic organization of the *Tanf_01300* locus is shown for the parent strain *T. forsythia* ATCC 43037, the Δ*Tanf_01300* mutant and the reconstituted mutant Δ*Tanf_01300*$^+$. Black coloured arrows represent primers used for PCR amplification of genes and homologous regions, red coloured primers represent those used to screen for correct integration of the knock-out and reconstitution cassettes; restriction sites used for cloning are indicated (not drawn to scale). **(B)** Agarose gel electrophoresis (left) confirms the deletion of *Tanf_01300* using the upstream primers 500/524 (1184 bp) and downstream primers 525/501 (1137 bp) on genomic DNA of *T. forsythia* ATCC 43037 Δ*Tanf_01300* mutant with integrated *ermF* cassette. Primers 506/507 yield a 224-bp PCR fragment when using *T. forsythia* wild-type genomic DNA, whereas this fragment is absent on genomic DNA of the Δ*Tanf_01300* mutant confirming the loss of the gene (log). Agarose gel electrophoresis (right) confirms the reconstitution of the deleted *Tanf_01300* gene using upstream primers 500/565 (2446 bp) and downstream primers 48/501 (1143 bp). Screening primers 500/501 yield in a 4184-bp PCR product on genomic DNA of the reconstituted mutant Δ*Tanf_01300*$^+$ (mut) with integrated *cat* resistance gene, whereas the same primer pair results in a 3527-bp product on genomic DNA of the *T. forsythia* wild-type. O´Gene Ruler 1 kb Plus DNA Ladder (Thermo Fisher Scientific) was used as a gene ladder.

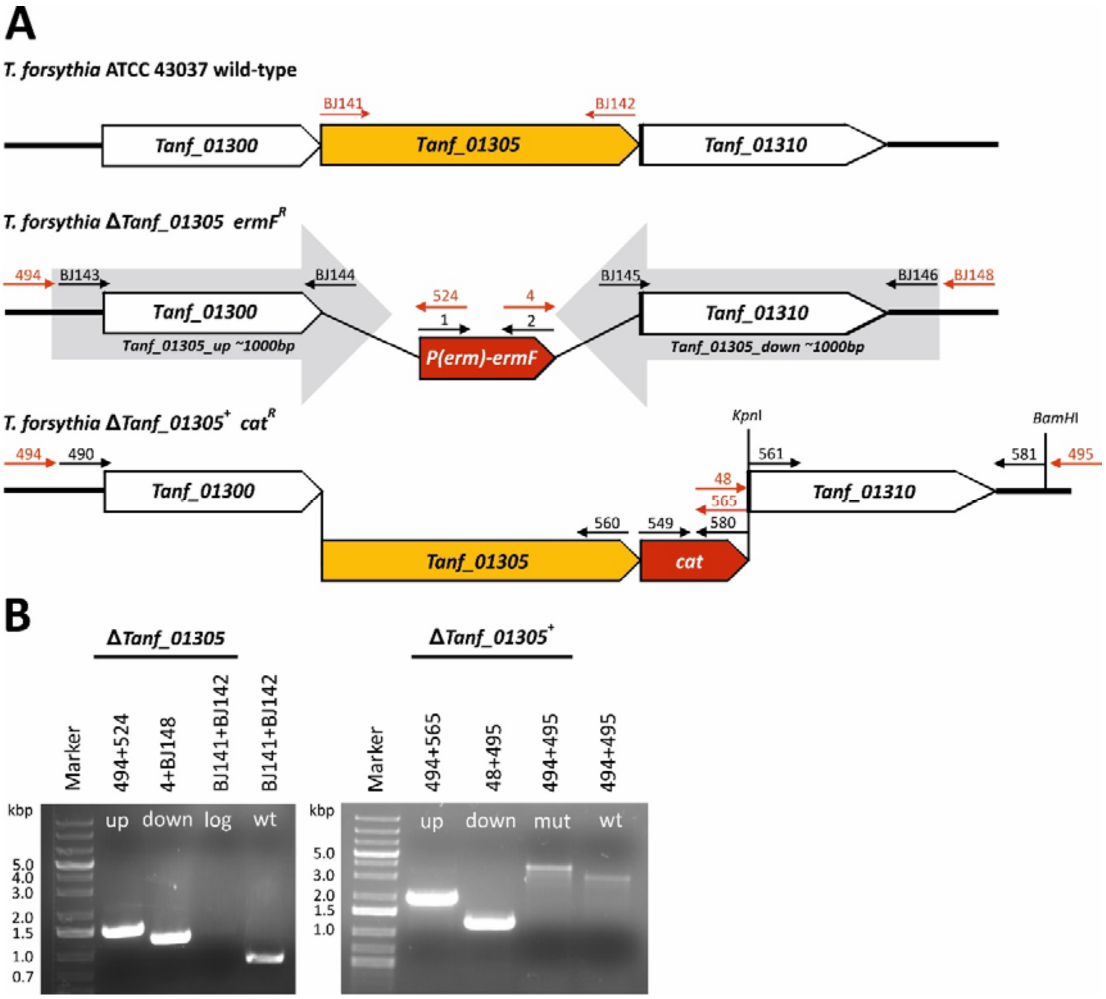

Supplementary FIGURE S5 | Strategy for the generation of a *T. forsythia* ATCC 43037 mutant at the *Tanf_01305* locus and confirmation by PCR. **(A)** The genomic organization of the *Tanf_01305* locus is shown for the parent strain *T. forsythia* ATCC 43037, the Δ*Tanf_01305* mutant and the reconstituted mutant Δ*Tanf_01305*$^{+}$. Black coloured arrows represent primers used for PCR amplification of genes and homologous regions, red coloured primers represent those used to screen for correct integration of the knock-out and reconstitution cassettes; restriction sites used for cloning are indicated (not drawn to scale). **(B)** Agarose gel electrophoresis (left) confirms the deletion of *Tanf_01305* using the upstream primers 494/524 (1432 bp) and downstream primers 4/BJ148 (1192 bp) on genomic DNA of *T. forsythia* ATCC 43037 Δ*Tanf_01305* mutant with integrated (P*erm*)-*ermF* cassette. Primers BJ141/BJ142 yield a 768-bp PCR fragment when using *T. forsythia* wild-type genomic DNA, whereas this fragment is absent on genomic DNA of the Δ*Tanf_01305* mutant confirming the loss of the gene (log). Agarose gel electrophoresis (right) confirms the reconstitution of the deleted *Tanf_01305* gene using upstream primers 494/565 (1799 bp) and downstream primers 48/495 (1179 bp). Screening primers 494/495 yield in a 3680-bp PCR product on genomic DNA of the reconstituted mutant Δ*Tanf_01305*$^{+}$ (mut) with integrated *cat* resistance gene, whereas the same primer pair results in a 3023-bp product on genomic DNA of the *T. forsythia* wild-type. O´Gene Ruler 1 kb Plus DNA Ladder (Thermo Fisher Scientific) was used as a gene ladder.

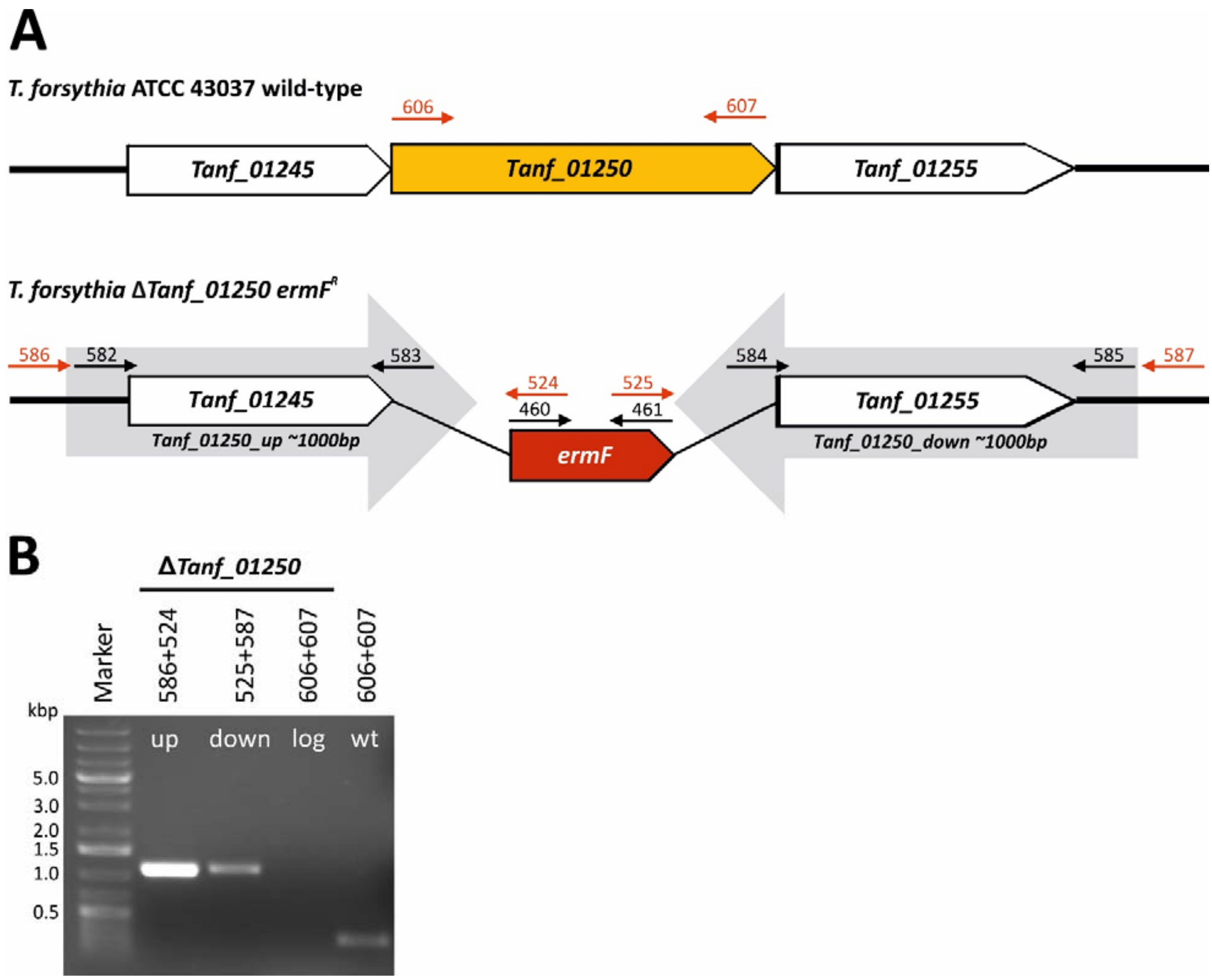

Supplementary FIGURE S6 | Strategy for the generation of a *T. forsythia* ATCC 43037 mutant at the *Tanf_01250* locus and confirmation by PCR. **(A)** The genomic organization of the *Tanf_01250* locus is shown for the parent strain *T. forsythia* ATCC 43037 and the Δ*Tanf_01250* mutant. Black coloured arrows represent primers used for PCR amplification of genes and homologous regions, red coloured primers represent those used to screen for correct integration of the knock-out and reconstitution cassettes; (not drawn to scale). **(B)** Agarose gel electrophoresis confirms the deletion of *Tanf_01250* using the upstream primers 586/524 (1108 bp) and downstream primers 525/587 (1122 bp) on genomic DNA of *T. forsythia* ATCC 43037 Δ*Tanf_01250* mutant with integrated *ermF* cassette. Primers 606/607 yield in a 273-bp PCR fragment when using *T. forsythia* wild-type genomic DNA (wt), whereas this fragment is absent on genomic DNA of the Δ*Tanf_01250* mutant confirming the loss of the gene (log). O´Gene Ruler 1 kb Plus DNA Ladder (Thermo Fisher Scientific) was used as a gene ladder.

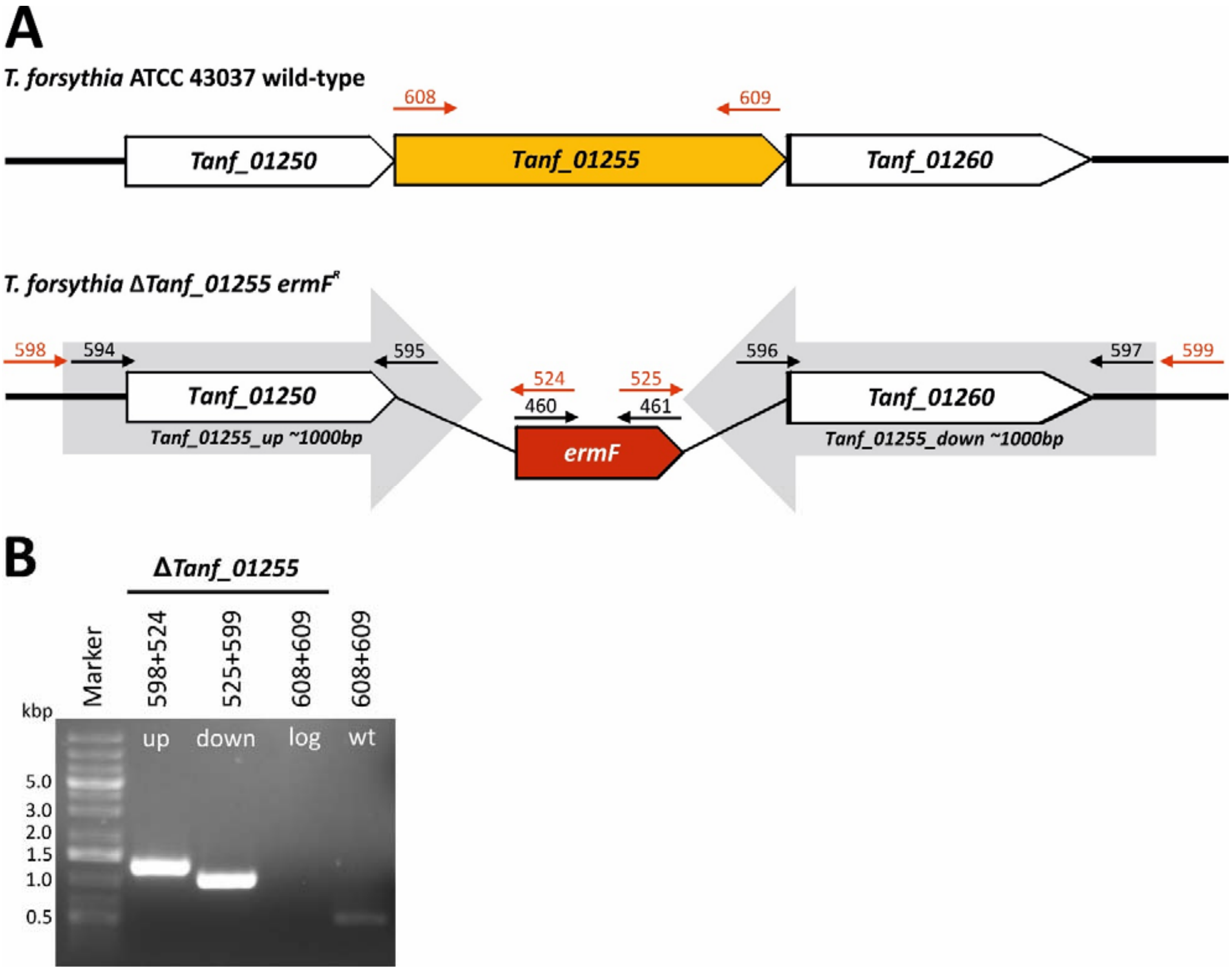

Supplementary FIGURE S7 | Strategy for the generation of a *T. forsythia* ATCC 43037 mutant at the *Tanf_01255* locus and confirmation by PCR. **(A)** The genomic organization of the *Tanf_01255* locus is shown for the parent strain *T. forsythia* ATCC 43037 and the Δ*Tanf_01255* mutant. Black coloured arrows represent primers used for PCR amplification of genes and homologous regions, red coloured primers represent those used to screen for correct integration of the knock-out and reconstitution cassettes; (not drawn to scale). **(B)** Agarose gel electrophoresis confirms the deletion of *Tanf_01255* using the upstream primers 598/524 (1234 bp) and downstream primers 525/599 (1029 bp) on genomic DNA of *T. forsythia* ATCC 43037 Δ*Tanf_01255* mutant with integrated *ermF* cassette. Primers 608/609 yield in a 504-bp PCR fragment when using *T. forsythia* wild-type genomic DNA (wt), whereas this fragment is absent on genomic DNA of the Δ*Tanf_01255* mutant confirming the loss of the gene (log). O´Gene Ruler 1 kb Plus DNA Ladder (Thermo Fisher Scientific) was used as a gene ladder.

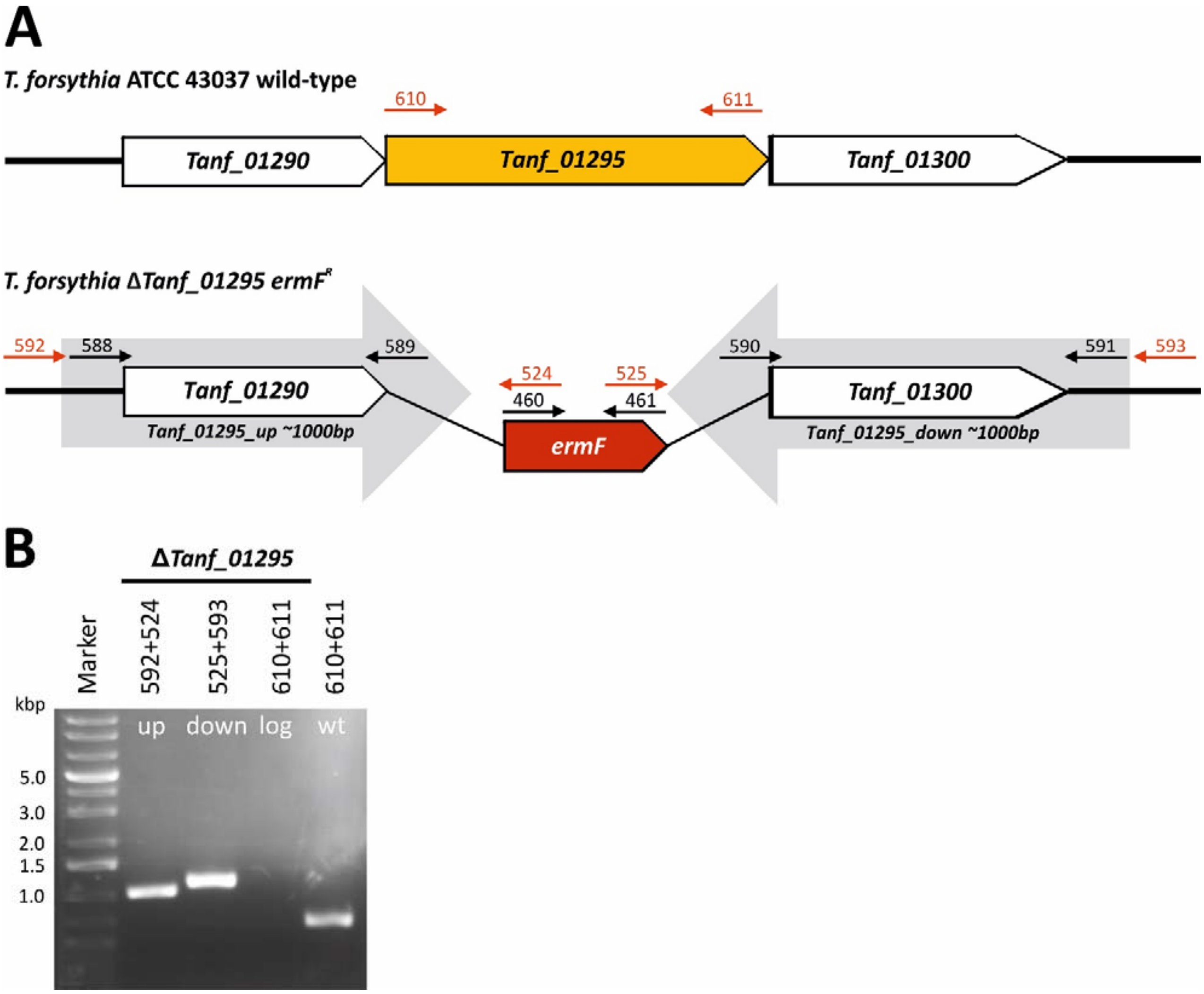

Supplementary FIGURE S8 │ Strategy for the generation of a *T. forsythia* ATCC 43037 mutant at the *Tanf_01295* locus and confirmation by PCR. **(A)** The genomic organization of the *Tanf_01295* locus is shown for the parent strain *T. forsythia* ATCC 43037 and the Δ*Tanf_01295* mutant. Black coloured arrows represent primers used for PCR amplification of genes and homologous regions, red coloured primers represent those used to screen for correct integration of the knock-out and reconstitution cassettes; (not drawn to scale). **(B)** Agarose gel electrophoresis confirms the deletion of *Tanf_01295* using the upstream primers 592/524 (1023 bp) and downstream primers 525/593 (1172 bp) on genomic DNA of *T. forsythia* ATCC 43037 Δ*Tanf_01295* mutant with integrated *ermF* cassette. Primers 610/611 yield in a 642-bp PCR fragment when using *T. forsythia* wild-type genomic DNA (wt), whereas this fragment is absent on genomic DNA of the Δ*Tanf_01295* mutant confirming the loss of the gene (log). O´Gene Ruler 1 kb Plus DNA Ladder (Thermo Fisher Scientific) was used as a gene ladder.

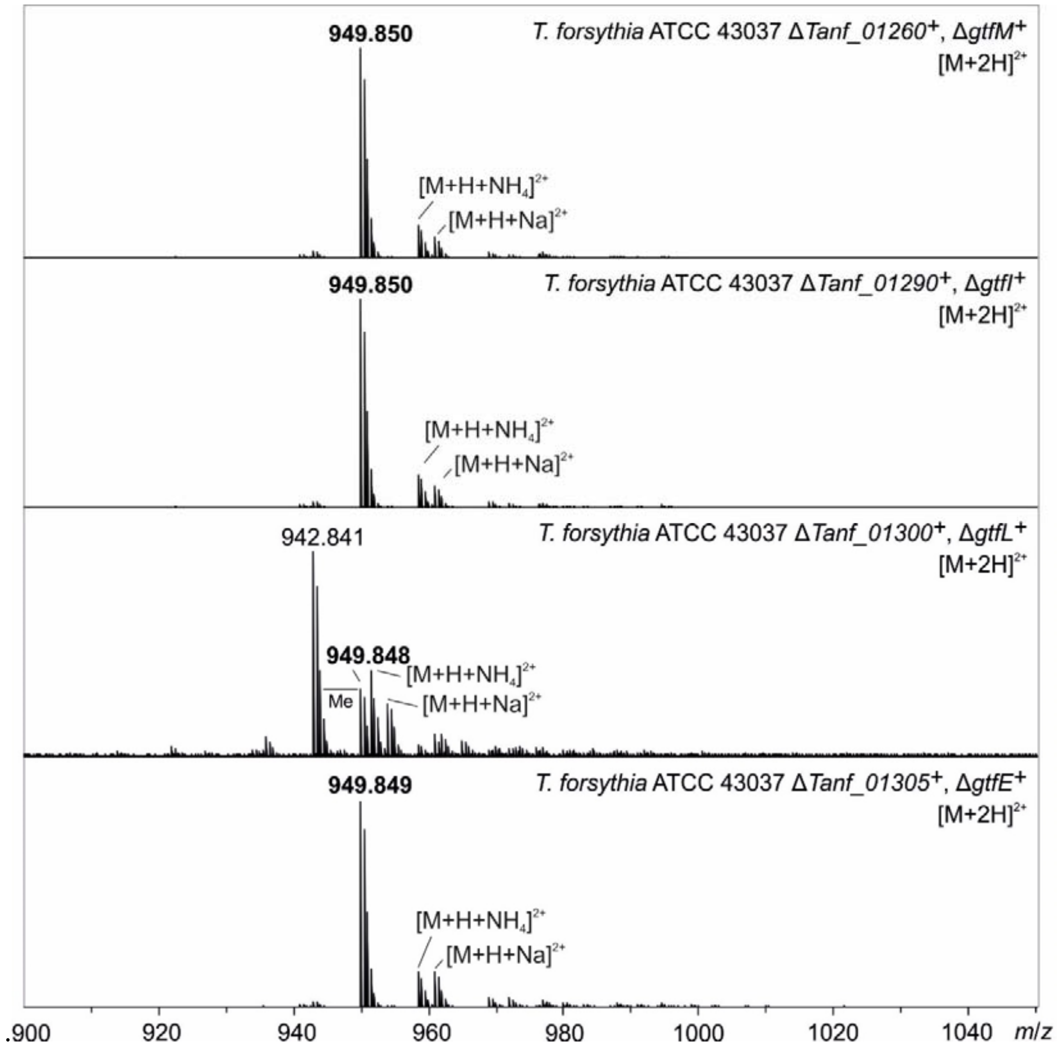

Supplementary FIGURE S9 | Deconvoluted Q-TOF-MS sum spectra of β-eliminated TfsB *O*-glycans from complemented *T. forsythia* ATCC 43037 glycosyltransferase-deficient strains (*T. forsythia* Δ*gtfM*$^+$, *T. forsythia* Δ*gtfI*$^+$, *T. forsythia* Δ*gtfL*$^+$, and *T. forsythia* Δ*gtfE*$^+$)**.** All samples were measured in positive mode with a quadrupole time-of-flight (Q-TOF) instrument (maXis 4G; Bruker, Bremen, Germany). Standard source settings (capillary voltage 4.5 kV, nebulizer gas pressure 0.5 bar, drying gas 5 l/min, 200°C) were used (Grünwald-Gruber et al., 2017). Instrument tuning was optimized for a mid-mass range (500–3000-Da molecules). The purified samples were loaded on a PGC column (100 mm x 0.32 mm, 5 µm; Thermo Scientific, Waltham, MA) with use of 65 mM ammonium formate buffer, pH 3.0, as the aqueous solvent (A). A gradient from 1% solvent B (80% acetonitrile plus 20% solvent A) to 68% solvent B in 40 min was applied, at a flow rate of 6 µl/min. Detection was performed in data-dependent acquisition mode (switching to MS/MS mode for eluted peaks), directly linked to a Thermo Ultimate 3000 UPLC system. MS scans were recorded (range 150–2200 *m*/*z*) and the four highest peaks were selected for fragmentation. Instrument calibration was performed with an ESI calibration mixture (Agilent). Data interpretation was done with DataAnalysis 4.0 (Bruker). Relative peak intensities of occurring peaks are given on the *y* axis.Data for *T. forsythia* Δ*gtfS*$^+$ can be found elsewhere (Tomek et al., 2017).

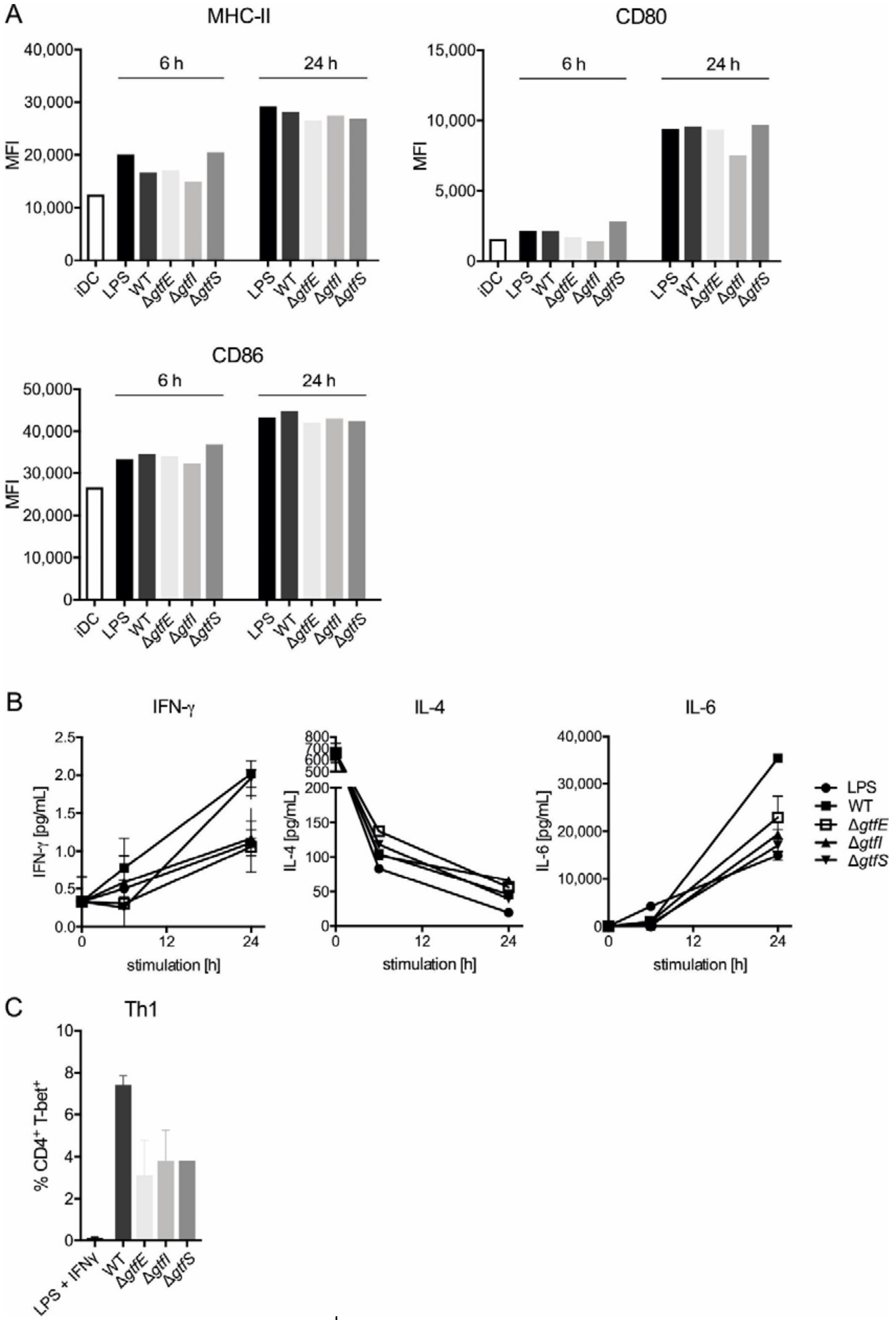

Supplementary FIGURE S10 |

Supplementary FIGURE S10 | Effects of *T. forsythia* protein glycosylation on human DC phenotype and functionality. (A) Surface expression of DC activation markers upon stimulation by *T. forsythia* wild-type (WT) and glycosyltransferase-deficient strains as compared to LPS and measured by flow cytometry. (iDCs, immature = unstimulated DCs) **(B)** Secretion of inflammatory cytokines by human DCs upon stimulation by *T. forsythia* wilt-type and glycosyltransferase-deficient strains as measured in culture supernatants by ProcartaPlex Multiplex Immunoassay (n=3). **(C)** T cell-priming upon antigen-presenting cell (APC) stimulation with *T. forsythia* wild-type and glycosyltransferase-deficient strains was assessed by culturing human peripheral blood mononuclear cells (PBMCs). Th1 differentiation was assessed by expression of the signature transcription factor T-bet as measured by flow cytometry (n=3). All data are presented as mean ±SEM.

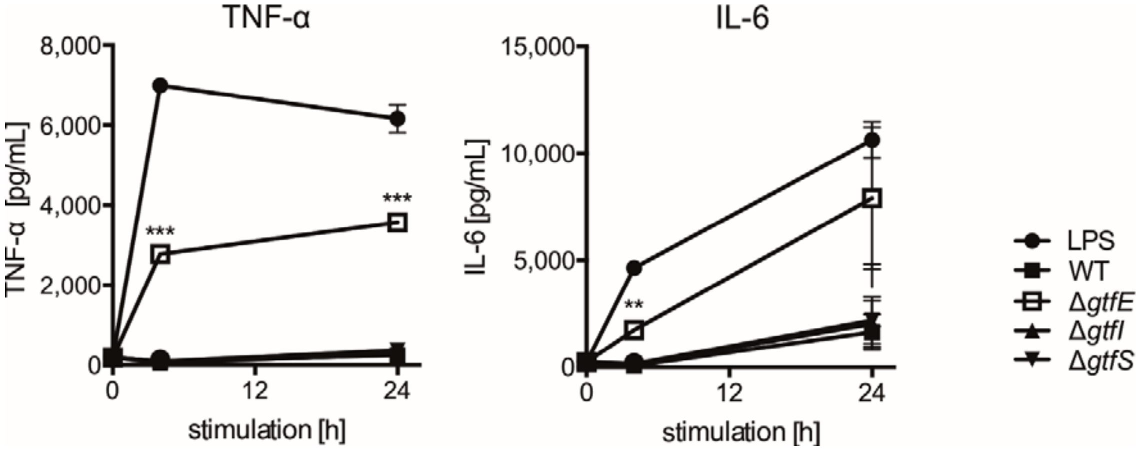

Supplementary FIGURE S11 | Secretion of inflammatory cytokines TNF-α and IL-6 by murine DCs upon stimulation with *T. forsythia* ATCC 43037 wild-type (WT) **and glycosyltransferase-deficient mutants** (*T. forsythia* Δ*gtfE*, Δ*gtfI*, and Δ*gtfS*) as measured in culture supernatants by ELISA. All data are presented as mean ± SEM of triplicate determinations. One representative out of three independent experiments is shown. Statistically significant differences are indicated as **$p<0.01$ and ***$p<0.001$ (unpaired Student's t-test). LPS, *E. coli* O111:B4 LPS.

Supplementary TABLE S1 | *Tannerella* strains used for genome sequence comparisons.

Strain/Species	Accession	Annotation date / source
T. forsythia FDC 92A2	NC_016610.1	18-08-2015 / RefSeq
T. forsythia ATCC 43037	NZ_JUET00000000.1	18-08-2015 / RefSeq
T. forsythia KS16	NZ_AP013045.1	04-02-2016 / RefSeq
T. forsythia 3313	NZ_AP013044.1	04-02-2016 / RefSeq
T. forsythia UB4	FMMN01000000.1	01-10-2016 / GenBank
T. forsythia UB20	FMMM01000000.1	01-10-2016 / GenBank
T. forsythia UB22	FMML01000000.1	01-10-2016 / GenBank
Tannerella sp. HOT-286	NZ_CP017038.1	04-10-2016 / RefSeq

Supplementary TABLE S2 | Oligonucleotide primers used for PCR amplification reactions.

Primers	Sequence (5´-3´)
490	GGGATATCGTATTGACCGGCATCCTC
500	GGAGATGGCCCTGAAAGACAAACACTG
527	TGCCCACTGGTCATGCATCG
528	AATCTTTCAGGAAATCCGTAGGATTTGGATA
529	ATGTCAGAGCCGGAAATGAAATTATCCGTG
531	GTAATAACCATATCTGCCTCTGGAAC
553	ATCTTAACCCGGACCCAACCGGATG
607	TCATGAATAGTCATCCATTTTAC
610	ATGTTTTTGACCGTTTTCTTCCCAAAG
611	TTATTTTCTCGCTGCAATCACAATATAG
642	ATGGATATAGGGCAAATAAGAAA
644	AAGTAATAGAACACGTACATAATG
645	ATGGGCATTTGATGTCAACTTTG
646	AAGACGGAATGGTTACATTTAC
648	CCGCAACGCTTCGCAACGTC
649	TTAAAATCTGGGAAATACAAG
650	AATAGCAGAGATAGACCCTAATG
651	AATGCGGTTGGACTTAGATTAC
652	CTCAATAACGGTCGTAGATTC
653	AATTAGTGCAGCAGGGACAG
653	AATTAGTGCAGCAGGGACAG
654	GGCCCGTTTGTATTTATCTC
655	AAGGGGTGCTGATGCGTGTAGG
656	GCTGCTTCGGTATTCACCGGGAAG
657	AGGTAAAGATGCCGACAGAG
658	TAAATGGCTCCTTCCAATTCC
659	GTCCGGCAAAGTATATTACCC
660	CCCGGTGATAAGCACAAG
661	ACATATTATTCTCTGCTAAG
662	CGTCTGTGATAGTAATCATTTC
663	GGCGGAATACATAAAGCATC
664	GTCGCCTGCGAATTATACCC
665	ATATGTTATTCAGGTAGAAG
666	CAGATAAGGAGCAAATTGTC
667	GACAAGCAATCCGCTCATAG
668	CCTTCATTGCGTCTGTATTG
669	TAATCAATACAGACGCAATG
670	TGCAGCGGTCGGACCAATTC
671	GCGGTAGTCGGTAAACGTC
672	CAGTTACCGAATAGTTTACTG
673	AGATTCTACCTCCAAAGAAG
674	CCGCCTGTTTATTTGATTCG
675	TAGAGATTCATCCTTAATAG
676	CAATCGCCTCTACGGGCTTG
677	ATTATTCAAGCCCGTAGAGG
678	TACTGTCTGTACTGATATTC
681	GACAGATGAAACTGCACAAAG
682	TGCAATTACGGGACATTTAC
694	ATCCTGCAGAACGTGCATTG
695	TATTTCTTCCGGTTCAAATG

Supplementary TABLE S3 | Amplification (black) and screening (red) oligonucleotide primers used for PCR reactions to generate *T. forsythia* ATCC 43037 glycosyltransferase knock-out mutants. Nucleotides used for OE-PCRs are written in bold, artificial restriction sites are underscored. Lowercase letters indicate artificially introduced bases for restriction endonuclease digestion.

Primers	Sequence (5´-3´)
**Amplification and screening primers for mutants at the *Tanf_01260* locus**	
IN475[a]	GATTTGAGAAAGATTAAATGGAAACAATATTTG
IN476[a]	**GTAAAACGAACGGGCAATTTCTTTTTTGTCAT**TTTAACTATTTTATTCAATCATAGTTTTCGATAC
IN477[a]	**GTTGTCCCTGAAAAATTTCATCCTTCGTAG**TAATAAGATGTGTGGAATAACAGGGTATATCTC
IN478[a]	TGGGCATTCAAATTATAATATTGCACAC
460[b]	ATGACAAAAAAGAAATTGCCCGTTCGTTTTAC
461[b]	CTACGAAGGATGAAATTTTTCAGGGACAAC
547	AAAGAGGATCTACGAACAATAAC
550	gactGGTACC GCATGCTTATAAAAGCCAGTCATTAGG
563	**TCCAATTGTCTAAATCAATTTTATTAAAGTTCATTGTACA**TTATAATTCTTTTATCCATTCTAATACATTTG
564	**GAATGGATAAAAGAATTATAA**TGTACAATGAACTTTAATAAAATTGATTTAGACAATTG
572	gctaGCATGCTAAGATGTGTGGAATAACAGGGTATATC
573	gctaGGTACCCCTCCACTAAGCAAGGCTCCAAC
48[c]	GTCAGATAGGCCTAATGACTGGC
510	GGGCGCTTGTGTTCCTTCC
511	TCTTCGCATAGTATCAAGAG
524[b]	GTAAAACGAACGGGCAATTTCTTTTTTGTCAT
525[b]	CCCTGAAAAATTTCATCCTTCGTAG
528	AATCTTTCAGGAAATCCGTAGGATTTGGATA
529	ATGTCAGAGCCGGAAATGAAATTATCCGTG
565[b]	CTAAATCAATTTTATTAAAGTTCAT
**Amplification and screening primers for mutants at the *Tanf_01290* locus**	
460[b]	ATGACAAAAAAGAAATTGCCCGTTCGTTTTAC
461[b]	CTACGAAGGATGAAATTTTTCAGGGACAAC
549	ATGAACTTTAATAAAATTGATTTAG
550	gactGGTACC GCATGCTTATAAAAGCCAGTCATTAGG
553	ATCTTAACCCGGACCCAACCGGATG
554	**TAAATCAATTTTATTAAAGTTCAT**TGTACATCATTGTTGTTTACTTATTAC
570	gctaGCATGCATAACAATCTGACCGATCGCTC

571	gctaGGTACCCGAACCACTTAGGCTGTTTCTC
IN458n	TGCTAAACATTTTCGGGATTGTTCCGAACTACG
IN464	**GTAAAACGAACGGGCAATTTCTTTTTTGTCAT**CTTTTAAGAAAGCTGAAAAGAGTTAACAATC
IN465n	**GTTGTCCCTGAAAAATTTCATCCTTCGTAG**ATAACAATCTGACCGATCGCTCTT
IN468	TGAGCAGGAGTATCTTCATCTTATTTTCTCGC
48[c]	GTCAGATAGGCCTAATGACTGGC
508	TCTCGATTAACGACGCGATG
509	TATGAAGCCTCTCCCATAAC
524[b]	GTAAAACGAACGGGCAATTTCTTTTTTGTCAT
525[b]	CCCTGAAAAATTTCATCCTTCGTAG
526	TCAAGATGGCTCCGCTTGTG
527	TGCCCACTGGTCATGCATCG
565[b]	CTAAATCAATTTTATTAAAGTTCAT

Amplification and screening primers for mutants at the *Tanf_01300* locus

460[b]	ATGACAAAAAAGAAATTGCCCGTTCGTTTTAC
461[b]	CTACGAAGGATGAAATTTTTCAGGGACAAC
496	CGATTAACGACGCGATGTGTTTCTC
497	**GTAAAACGAACGGGCAATTTCTTTTTTGTCAT**CTTATTTCTCGCTGCAATCACAATATAG
498	**GCTGGAAGTTGTCCCTGAAAAATTTCATCCTTCGTAG**TATATGTCTCCTCTGTTTTCCATTATC
499	CGTGCTCTCCCGTTTCTTTATAGTAATC
549	ATGAACTTTAATAAAATTGATTTAG
557	**CTAAATCAATTTTATTAAAGTTCATTGTACATT**ATGACAATAGACTGTGATATAATG
558	gcatGGTACCTATATGTCTCCTCTGTTTTCCATTATC
574	gctaCATATGCGTGCTCTCCCGTTTCTTTATAGTAATC
BJ127	aatcaCATATG GGTACCTTATAAAAGCCAGTCATTAGGCCTATCTGAC
48[c]	GTCAGATAGGCCTAATGACTGGC
500	GGAGATGGCCCTGAAAGACAAACACTG
501	CCGGAAAGGAAGTAGAGCGAGAACAC
506	GCAGCCTTATTGCCTTATCC
507	TCTGCCACATACCCGTTCTG
524[b]	GTAAAACGAACGGGCAATTTCTTTTTTGTCAT
525[b]	CCCTGAAAAATTTCATCCTTCGTAG
565[b]	CTAAATCAATTTTATTAAAGTTCAT

Amplification and screening primers for mutants at the *Tanf_01305* locus

1[c]	GGTACCCCCGATAGCTTCCGCTATTGC
2[c]	CTACGAAGGATGAAATTTTTCAGGG
490	GGGATATCGTATTGACCGGCATCCTC
549	ATGAACTTTAATAAAATTGATTTAG
560	**GTCTAAATCAATTTTATTAAAGTTCATTGTACA**CTAATAATTGTTCCCGAAAAGCTTCG
561	gtcaGGTACCTGAATCGACATTATAATGTAAGAATTG
580	aatcaGGATCC GGTACCTTATAAAAGCCAGTCATTAGGCCTATCTGAC
581	gatcGGATCCATCGTAATGCGAGCTGGCTCCGTAG
BJ143	GGATATCGTATTGACCGGCATCCTCTC
BJ144	**GCAATAGCGGAAGCTATCGGGGGTACC**CATATTATGACAATAGACTGTGATATAATG
BJ145	**CCCTGAAAAATTTCATCCTTCGTAG**TGAATCGACATTATAATGTAAGAATTGAAAAGATAG
BJ146	CTGTCCGTTACGGCTGAAGAAATGG
4[c]	CCCTGAAAAATTTCATCCTTCGTAG
48[c]	GTCAGATAGGCCTAATGACTGGC
494	CAGCCTAAGTGGTTCGGCAAGTTC
495	CATCGTTCGGGCCCTTGCACGATAAGC
524[b]	GTAAAACGAACGGGCAATTTCTTTTTTGTCAT
565[b]	CTAAATCAATTTTATTAAAGTTCAT
BJ141	ATGTCTCCTCTGTTTTCCATTATCACC
BJ142	CTAATAATTGTTCCCGAAAAGCTTCGCCC
BJ148	CACGGCAAGCAACCACCATCGTTC

[a] Sequence (3'-5'); [b] (Tomek et al., 2017); [c] (Friedrich et al., 2017)

Supplementary TABLE S4 | Amplification (black) and screening (red) oligonucleotide primers used for PCR reactions to generate methyltransferase knock-out mutants. Nucleotides used for OE-PCRs are written in bold.

Primers	Sequence (5´-3´)
**Amplification and screening primers for a mutant at the *Tanf_01250* locus**	
582	GACTGTAGATGCGTATCCACAATTTC
583	**AAACGAACGGGCAATTTCTTTTTTGTCAT**TGTACCTCTATACTTATATTACTGTTATTG
584	**TGTCCCTGAAAAATTTCATCCTTCGTAG**ATTTTCATTTCCTGAATTAAAGTAC
585	ATAACCATATCTGCCTCTGGAAC
460[a]	ATGACAAAAAAGAAATTGCCCGTTCGTTTTAC
461[a]	CTACGAAGGATGAAATTTTTCAGGGACAAC
586	CGAGACCCTACCTGAATTTGTATG
587	TGGTATCTTCTTTGTATAAC
524[a]	GTAAAACGAACGGGCAATTTCTTTTTTGTCAT
525[a]	CCCTGAAAAATTTCATCCTTCGTAG
606	ATGGATATAGGGCAAATAAG
607	TCATGAATAGTCATCCATTTTAC
**Amplification and screening primers for a mutant at the *Tanf_01255* locus**	
594	CTGTCGCATTCCCTGGAGATACAAG
595	**CGGGCAATTTCTTTTTTGTCAT**CCATTTTACCCCCCCCCTGAAAAC
596	**GTCCCTGAAAAATTTCATCCTTCGTAG**ATAAAATAGTTAAATGAGGAAAATC
597	CATCACCTAATGGTATAGAAGCTAAC
460[a]	ATGACAAAAAAGAAATTGCCCGTTCGTTTTAC
461[a]	CTACGAAGGATGAAATTTTTCAGGGACAAC
598	GACTGTAGATGCGTATCCACAATTTC
599	CAGCTCTTCCATAGTTATTCTC
524[a]	GTAAAACGAACGGGCAATTTCTTTTTTGTCAT
525[a]	CCCTGAAAAATTTCATCCTTCGTAG
608	ATGACTATTCATGAATTTTCATTTC
609	TCAATCATAGTTTTCGATACAT

Amplification and screening primers for a mutant at the *Tanf_01295* locus

588	CGGAAAGCTGGGCTATTTCAAG
589	**CGGGCAATTTCTTTTTTGTCAT**CTTCTGCGGTACTTTTCCGTATTG
590	**CCCTGAAAAATTTCATCCTTCGTAG**GATGAAGATACTCCTGCTCAGTACGTTTGAG
591	TTATCTTCCATCGAAGGAATAAC
460[a]	ATGACAAAAAAGAAATTGCCCGTTCGTTTTAC
461[a]	CTACGAAGGATGAAATTTTTCAGGGACAAC
592	TTGACCGGAGTATTCTCAAAC
593	TTCTGCCACATACCCGTTCTG
524[a]	GTAAAACGAACGGGCAATTTCTTTTTTGTCAT
525[a]	CCCTGAAAAATTTCATCCTTCGTAG
610	ATGTTTTTTGACCGTTTTCTTCCCAAAG
611	TTATTTTCTCGCTGCAATCACAATATAG

[a] (Tomek et al., 2017)

Supplementary TABLE S5 | Locus tags for S-layer genes of *T. forsythia* strains and isolate *Tannerella* sp. BU063.

**Strain**	***tfsA***<br>*locus tag*<br>GenBank accession	***tfsB***<br>*locus tag*<br>GenBank accession
**ATCC 43037**	*Tanf_03370*<br>KKY61889.1	*Tanf_03375*<br>KKY61890.1
**UB20**	*TFUB20_01413*<br>SCQ21558.1	*TFUB20_01414*<br>SCQ21562.1
**FDC 92A2**	*BFO_1650-1* (*TF2661-2*)<br>AAR82922	*BFO_1652* (*TF2663*)<br>AEW21150
**UB4**	*TFUB4_01362*<br>SCQ20752.1	*TFUB4_01363*<br>SCQ20755.1
**KS16**	*TFKS16_1455*<br>BAR51709.1	*TFKS16_1457*<br>BAR51711.1
**UB22**	*TFUB22_01373*<br>SCQ22347.1	*TFUB22_01374*<br>SCQ22351.1
**3313**	*TF3313_1507*<br>BAR49026.1	*TF3313_1509*<br>BAR49028.1
**BU063**	*BCB71_00675*<br>AOH39800.1	*BCB71_00680*<br>AOH39801.1

Supplementary References

Friedrich, V., Janesch, B., Windwarder, M., Maresch, D., Braun, M.L., Megson, Z.A., Vinogradov, E., Goneau, M.F., Sharma, A., Altmann, F., Messner, P., Schoenhofen, I.C., and Schäffer, C. (2017). *Tannerella forsythia* strains display different cell-surface nonulosonic acids: biosynthetic pathway characterization and first insight into biological implications. *Glycobiology* 27, 342-357. doi: 10.1093/glycob/cww129

Grünwald-Gruber, C., Thader, A., Maresch, D., Dalik, T., and Altmann, F. (2017). Determination of true ratios of different *N*-glycan structures in electrospray ionization mass spectrometry. *Anal. Bioanalyt. Chem*. 409, 2519-2530. doi: 10.1007/s00216-017-0235-8

Tomek, M.B., Janesch, B., Maresch, D., Windwarder, M., Altmann, F., Messner, P., and Schäffer, C. (2017). A pseudaminic acid or a legionaminic acid derivative transferase is strain-specifically implicated in the general protein *O*-glycosylation system of the periodontal pathogen *Tannerella forsythia*. *Glycobiology* 27, 555-567. doi: 10.1093/glycob/cwx019
